# Government plans in the 2016 and 2021 Peruvian presidential elections: A natural language processing analysis of the health chapters

**DOI:** 10.12688/wellcomeopenres.16867.1

**Published:** 2021-07-08

**Authors:** Rodrigo M. Carrillo-Larco, Manuel Castillo-Cara, Jesús Lovón-Melgarejo

**Affiliations:** 1 Department of Epidemiology and Biostatistics, Imperial College London, London, SW7 2AZ, UK; 2 CRONICAS Centre of Excellence in Chronic Diseases, Universidad Peruana Cayetano Heredia, Lima, Peru; 3 Universidad de Lima, Lima, Peru; 4 Paul Sabatier University, Toulouse, France

**Keywords:** Public health, health policy, Natural Language Processing, Latin America and the Caribbean, Peru, COVID-19

## Abstract

**Background**: While clinical medicine has exploded, electronic health records for Natural Language Processing (NLP) analyses, public health, and health policy research have not yet adopted these algorithms. We aimed to dissect the health chapters of the government plans of the 2016 and 2021 Peruvian presidential elections, and to compare different NLP algorithms

**Methods**: From the government plans in Peru (18 in 2016; 19 in 2021) we extracted each sentence from the health chapters. We used five NLP algorithms to extract keywords and phrases from each plan: Term Frequency – InverseDocument Frequency (TF-IDF), Latent Dirichlet Allocation (LDA), TextRank, Keywords Bidirectional Encoder Representations from Transformers (KeyBERT), and Rapid Automatic Keywords Extraction (Rake).

**Results**: In 2016 we analysed 630 sentences, whereas in 2021 there were 1,685 sentences. The TF-IDF algorithm showed that in 2016, nine terms appeared with a frequency of 0.10 or greater, while in 2021 43 terms met this criterion. The LDA algorithm defined two groups. The first included terms related to things the population would receive (e.g., ’insurance’), while the second included terms about the health system (e.g., ’capacity’). In 2021, most of the government plans belonged to the second group. The TextRank analysis provided keywords showing that ’universal health coverage’ appeared frequently in 2016, while in 2021 keywords about the COVID-19 pandemic were often found. The KeyBERT algorithm provided keywords based on the context of the text. These keywords identified some underlying characteristics of the political party (e.g., political spectrum such as left-wing). The Rake algorithm delivered phrases, in which we found ’universal health coverage’ both in 2016 and 2021.

**Conclusions**: NLP analysis could be used to inform on the underlying priorities in each government plan. NLP analysis could also be included in research of health policies and politics during general elections and provide informative summaries for the general population.

## Introduction

Over the last few years, researchers working in clinical medicine have adopted artificial intelligence and deep learning techniques such as Natural Language Processing (NLP). Due to this, electronic health records have become unique data sources because they contain free text annotations that can inform NLP models for a variety of tasks ^1– 7^ (e.g., risk prediction). On the other hand, public health research appears not to have benefited from NLP algorithms, despite the fact that this research field also has large data sources of text, such as reports and policy briefs ^8^. In this line, health policy research could also use NLP algorithms to scrutinize laws and other documents ^9^, such as health-related government plans and proposals during presidential election periods. This could help identify patterns within and between plans, to study underlying topics and key words or ideas, and to assess coherency within the text. Furthermore, following the global call to have evidence-based health policies, NLP algorithms could also help to study government plans in the context of the scientific literature (e.g., SciBERT) ^10, 11^. Overall, NLP could offer novel and insightful ways to dissect health-related government plans, so that practitioners, researchers, and public health experts have further arguments to imagine the future scenario of the health sector in their country. Similarly, the general population could benefit from this NLP analysis to compare across political parties and make a much more informed vote. Consequently, we aimed to describe the health chapters of the government plans in the ongoing (April 2021) and last (2016) presidential elections in Peru. In addition, we compared and discussed different NLP algorithms ^12– 14^. Finally, we have prepared and made available a toolkit so that others could replicate this work with their own texts or government plans. This work sought to introduce NLP into the health policy research agenda, while providing a new way of seeing, reading, and understanding the health chapters of government proposals during general elections.

## Methods

### Study design

This is an analysis of government plans from the 2016 and 2021 Peruvian presidential elections. For this analysis we used NLP algorithms: Term Frequency – Inverse Document Frequency (TF-IDF) ^15^, Latent Dirichlet Allocation (LDA) ^16^, TextRank ^17^, Keywords Bidirectional Encoder Representations from Transformers (KeyBERT) ^18^ and Rapid Automatic Keywords Extraction (Rake) ^19^.

In this analysis, we focused on the government plans regardless of the political party. That is, we do not make specific references or conclusions about the political parties; however, the names of the parties included in the study are presented in the tables and figures. We do not make specific arguments about the political parties to avoid introducing political bias or preferences. We aimed to make this a data-drive analysis to dissect the content of the government plans, and not a quality assessment of the government plans (or the political parties behind it).

### Scenario

According to the World Bank, Peru is an upper-middle income country in South America with a total population of 32.5 million people ^20^. Peru is a unitary presidential republic, in which the president is elected in general elections every five years; the most recent presidential elections took place in 2016, and the next one will take place in April 2021. The campaign for the forthcoming presidential elections started in December 2020 with over 15 candidates (i.e., political parties). Each candidate must publish a government plan.

### Data sources

In this study, we analysed the government plans of the 2016 and 2021 presidential elections in Peru. These plans are open access and in the public domain, so that all citizens can read these and become informed of their proposals ^21, 22^. For this work, which is health-oriented, we only selected the health chapters.

For the 2016 presidential elections, there were 19 government plans presented, although we analysed only 18 in this study as one was only available as a scanned image and could not be analysed as text. For the 2021 presidential elections, there were 20 government plans and we analysed just 19 as one was only available as a scanned image and could not be analysed as text. As we aimed to analyse the government plans, and not the political party, we analysed all government plans regardless of whether the party or the candidate was disqualified during the campaign period.

Within the health chapter of each government plan, we copied and pasted onto a spreadsheet each sentence in a row; in other words, for each government plan, we had as many rows as sentences. Furthermore, the dataset we generated had three columns: the name of the political party, a sentence indicator (e.g., political_party_A_sentence_1), and the sentences we extracted. All sentences were copied as they were in the original government plan. The government plans were translated into English using Google translate.

### Natural Language Processing Analysis

***Data preparation.*** NLP is a branch of artificial intelligence which aims to process and understand the human language. Not only does NLP need to understand the text (words), but it also needs to make sense of the context to provide accurate meaning to the text. To begin with NLP, we need to put a text into a two-dimensional matrix. In the model *Bag of Words* ^23^, each text or document is represented by the group of words in it.

As a previous step to the construction of the data matrix, a vocabulary is elaborated with the union of all the terms that appear in a document. From this, a matrix is built in which each row represents a document, and each column (each characteristic) corresponds to a term.

For this analysis we conducted a simple pre-processing, which included deleting HTML labels and non alphabetic characters for all datasets. Moreover, an advanced preprocessing of the datasets depends on the algorithm used. Therefore, in the description of the algorithms, the data pre-processing that has been used will be described for each algorithm.

***Algorithms***

**TF-IDF**: This is the most basic NLP algorithm. Each position ( *d*, *t*) in the matrix has a value for the metric *Term Frequency* which is denoted as *t f _d_*_, *t*_, and reflects the number of times the term *t* appears in the document *d*. There are variants to the *Term Frequency* metric, so that the matrix can include:

• *t f*_*d,t*_ ∈ {0,1} - if the term appears or not in the document.
• *log* (1 + *t f*_*d,t*_) - logarithmic scale.
• *t f*_*d,t*_/ *max*_*j*_ *t f* *_d,t_* - scale in relation to the most frequently used term in the document.

There are terms which appear several times in a document, but which not carry much information; these could include ’the’, ’a’, ’an’, and ’of’, among others. The metric *Inverse Document Frequency* penalises the terms in a magnitude equal to the frequency in which they appear in the text. The inverse frequency of a term is computed as:

$$idf_{t}=log\left(\frac{\#\;documents}{\#\;documents\;that\;include\;word\;t}\right)$$

Because both metrics ( *Term Frequency* and *Inverse Document Frequency*) provide information to the NLP algorithm to dissect the text, it is common practice to combine them as the TF-IDF metric, which can be expressed as:

$$t\;f-id\;f_{d,t}=t\;f_{d,t}\times id\;f_{t}$$

For TF-IDF, we used the dataset partitioned by phrases to analyse globally because a wide corpus of words is needed, so here the frequency of words in general is analysed (for all political parties).

Hence, we used the TfidfVectorizer class from the Scikit-Learn library with the parameter stop_words=’english’ ^24^; this parameter reads all the text from each government plan and removes non-relevant terms from a text such as ’the’ and ’as’, among others. Moreover, lemmatisation was used with the class WordNetLemmatizer from nltk library ^25^.

**LDA**: LDA builds a generative model from a set of observations that belong to unobserved groups. Basically, it considers each group as a probability distribution over the characteristics, and each observation as generated from a mixture of the groups’ probability distributions.

This model is frequently used to categorise a document into subjects or topics. For each topic, this model estimates the probability of the terms, while for the document, this model estimates the relevance of each topic.

Therefore we used LDA to define and describe, through the most relevant terms, the categories in which each data entry can be assigned to. We trained the LDA model with a dataset in which each sentence was an instance, and the categorisation was then conducted for the government plan.

We used LatentDirichletAllocation class from the Scikit-Learn library ^26^ with the parameter n_components=2; this parameter reads the number of topics to be created. For this work, we defined two groups (i.e., we specified that the algorithm should create two groups following an unsupervised approach from the data). Finally, for the data pre-processing, we used *stop_words* and *WordNetLemmatizer* classes from nltk library ^25^.

**TextRank:** This algorithm is a graph-based algorithm and it is based on *PageRank* ^17^ developed by Google, where PageRank is used to define the position of websites in the search engine through the extraction of key terms.

TextRank informs about the semantic sequence used in the text with unique keywords. We used the library Gensim ^27^.

For this analysis, we used the full government plans to extract the key words in each plan. Following the recommendations in the documentation for TextRank, we did not pre-proccess the data and thus used the original text as it was.

**KeyBERT:** KeyBERT ^12, 28^ is an adapted keyword extraction tool based on BERT ^18^. In the last couple of years, algorithms based on BERT are pushing the boundaries of the state of the art technology in social and technical multiple aspects of the natural language processing tasks ^29^?]. BERT is a pre-trained model on the Book-Corpus and English Wikipedia datasets ^30^. It uses a multi-head attention mechanism, so it can create better representations for entities considering the context, and leveraging the classic word-based approach.

For this analysis we used the full government plans to extract the keywords in each one. Also following the documentation for the KeyBERT algorithm, the texts were not pre-processed and used as they were. 

**Rake:** Rake ^14^ is used to extract keywords and key phrases. To use the Rake algorithm, we followed these steps:

1. In the text we identified stop words and punctuation.
2. We removed stop words and punctuation, and generated a list of the phrases that were separated by them.
3. We calculated the number of times each word appeared in all the phrases (i.e., frequency of a given word).
4. For two given different words in the text, we estimated how many times they were together in the same phrase (ie., a metric of co-occurrence).
5. For every given word, we calculated a score: frequency (step 3) divided by the co-occurrence (step 4).
6. A score for a complete given phrase was computed as the sum of the scores (step 5) of each word in such phrase.

For this analysis we used the full government plans to extract the phrases in each one. Also following the documentation for the Rake algorithm, the texts were not pre-processed and used as they were.

***Ethics.*** The underlying data for this study is accessible in the public domain and did not include the names of individuals, but the name of the political parties which created each government plan; this information is also in the public domain. Therefore, we considered this work of minimal risk and did not seek approval by an ethics committee or institutional review board.

## Results

### Data characteristics

The original dataset with the 2016 government plans had 559 rows (i.e., sentences) in total, which represented 18 government plans; the shortest government plan contributed with four sentences, while the longest contributed with 96 sentences (See Table 1). The original dataset with the 2021 government plans had 1,586 rows (i.e., sentences) in total, which represented 19 government plans; the shortest government plan had 10 sentences, while the longest had 215 sentences (See Table 1).

**Table 1.** Characteristics (number of sentences) of the original 2016 and 2021 datasets. Symbol — means that the political party did not stand for election in that year.

Political Party	Dataset 2016	Dataset 2021
Acción Popular	8	18
Alianza para el Progreso	6	95
Alianza Popular	33	—
APRA	—	84
Avanza Pais	—	10
Democracia Directa	4	40
Frente Amplio	67	83
Frente Esperanza	22	35
Fuerza Popular	25	122
Juntos por el Peru	—	124
Orden	74	—
Partido Humanista Peruano	7	—
Partido Morado	—	165
Partido Nacionalista Peruano	42	98
Partido Popular Cristiano	—	138
Perú Libertario	15	—
Peru Libre	—	55
Perú Nación	6	—
Perú Patria Segura	96	102
Perú Posible	23	—
Peruanos por el Kambio	94	—
Podemos	—	32
Progresando Perú	6	—
Renovación Popular	—	29
Siempre Unidos	20	—
Solidaridad Nacional – UPP	11	—
Somos Perú	—	215
Unión por el Perú	—	44
Victoria Nacional	—	97

### TF-IDF

**Figure 1.**
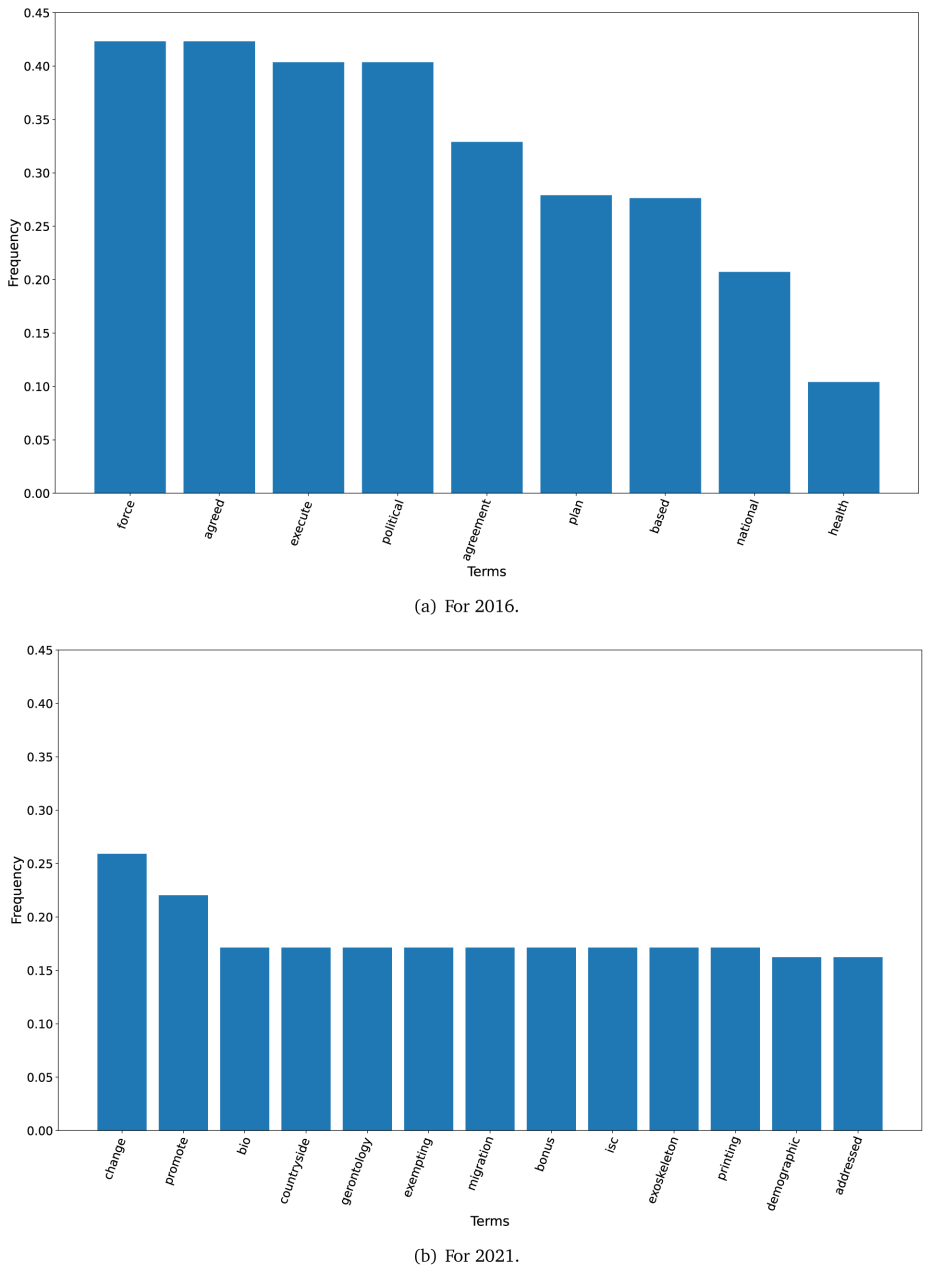
Frequent terms in the government plans (2016 and 2021) as per the TF-IDF algorithm.

The TF-IDF analysis showed differences between the plans in 2016 and 2021. When we set a threshold of 0.10 to select terms (i.e., terms that represented 10 per cent or more of all the words in the text), nine terms met this criterion in 2016 although 43 terms met this criterion in 2021. These terms are shown in Figure 1 for the years 2016 and 2021.

In 2016, across all government plans, the terms ’force’ and ’agreed’ had a TF-IDF frequency above 0.40, while the term ’health’ had a frequency of 0.10 (see Figure 1(a)).

In 2021, across all government plans, the term ’change’ had a TD-IDF frequency of 0.25, while the term ’demographic’ had a frequency of about 0.15 (see Figure 1(b)). Of note, for an accurate visual representation of Figure 1(b), we changed the frequency threshold from 0.10 to 0.156.

### LDA

The LDA algorithm was trained in a dataset with as many rows as sentences per government plan, to define two groups of ten words each (see Table 2); we did not define more groups, or more terms per group, because the dataset was small. For the prediction phase, we used the full text of each government plan (i.e., a dataset with as many rows as sentences per government plans), and analysed which group each government plan would belong. We did this analysis for the years 2016 and 2021 separately.

**Table 2.** Keywords classified to each group by LDA for each Government plan year.

Government plan year	Group 0	Group 1
**2016**	health public system improvised insurance service population private medicine national	health care disease quality program community access center hospital child
**2021**	health public system service insurance private access sector financing budget	health care national management population pandemic disease hospital capacity covid

Overall, in both 2016 and 2021, Group 0 appeared to cluster terms signaling things the population would receive (see Table 2). For example in 2016 and in 2021, Group 0 included ’service’ and ’insurance’. Conversely, in both 2016 and 2021, Group 1 appeared to cluster terms that are related with the structure of the health system like ’program’, ’hospital’, and ’capacity’ (see Table 2). Notably, ’covid’ appeared in Group 1 in 2021.

After generating the two groups (Group 0 and Group 1), we then answered: *to which group would each government plan belong?*

**Figure 2.**
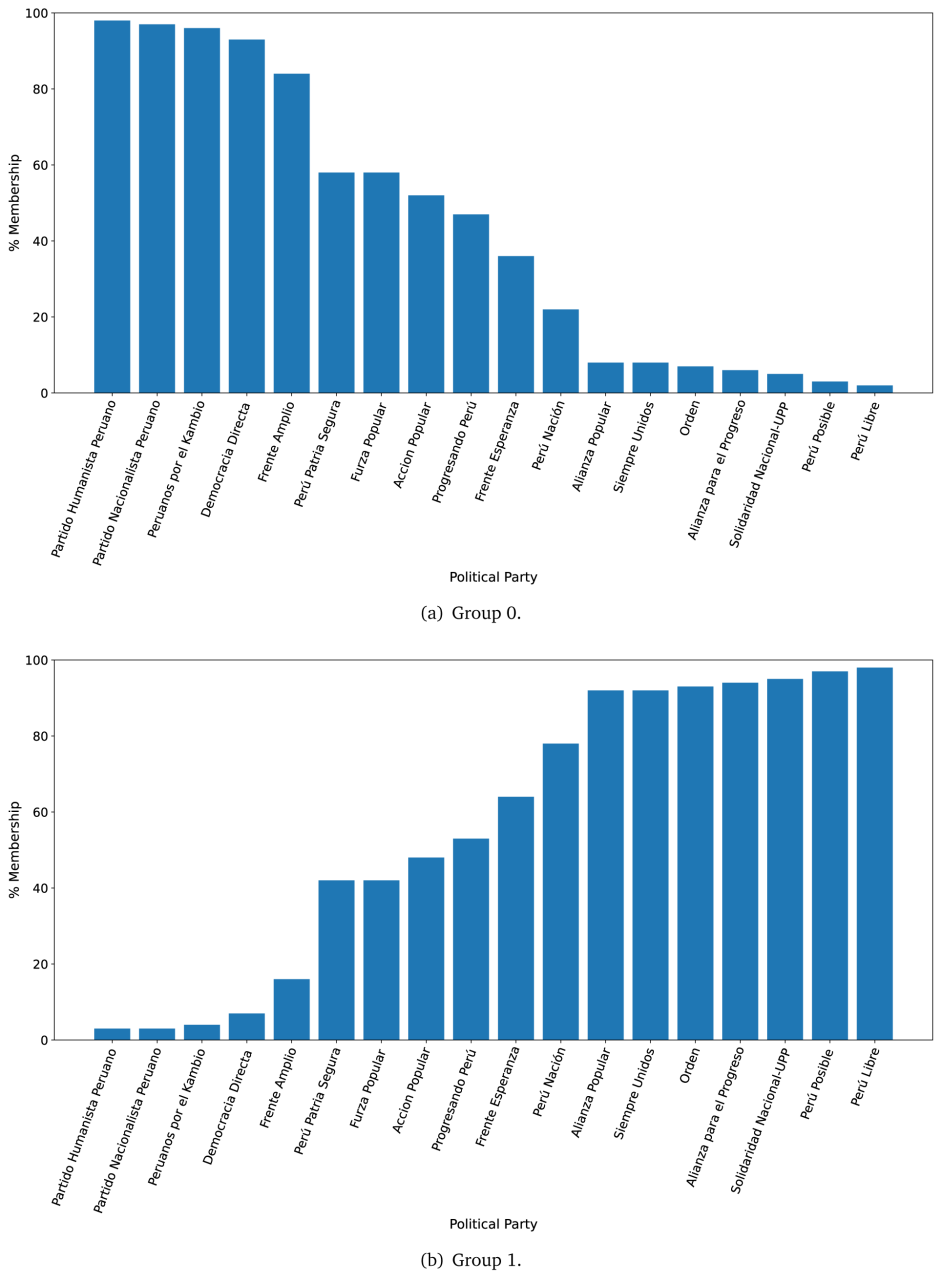
Membership of the 2016 government plans to each group classified by the LDA algorithm.

For the year 2016, we observed that the government plans likely to belong to Group 0, were much less likely to belong to Group 1 (see Figure 2(a), Figure 2(b)). There were four government plans with a probability between 0.40 and 0.60 of belonging to either Group 0 or Group 1. There was strong evidence suggesting that seven (out of eighteen) government plans would belong, almost exclusively, to Group 1 (see Figure 2(a), Figure 2(b)).

**Figure 3.**
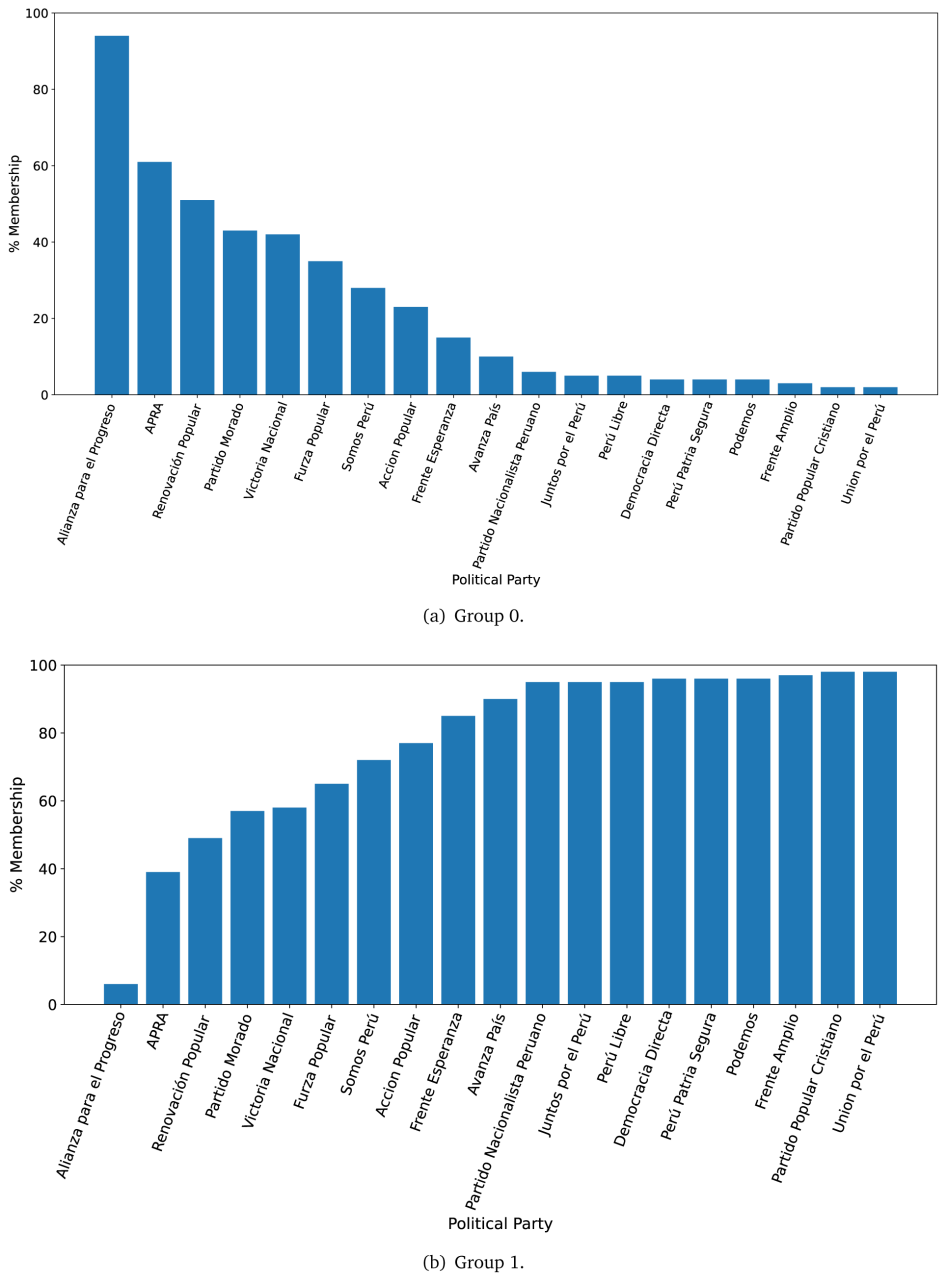
Membership of the 2021 government plans to each group classified by the LDA algorithm.

The distinction in favour of Group 0 was less clear when analysing the 2021 government plans (see Figure 3(a), Figure 3(b)), when we observed one government plan very likely to belong to Group 0 (in 2016 there were four). Conversely, there were nine government plans with high probability of belonging to Group 1, yet very low probability of belonging to Group 0.

### TextRank

The TextRank algorithm shows the keywords in the text. The number of keywords depends on the size and coherence of the text; that is, longer texts and those with more complexity would have more keywords than small texts with poor context. For an informative representation, we chose the top six keywords per government plan. Keyword could have between one and three words.

**Figure 4.**
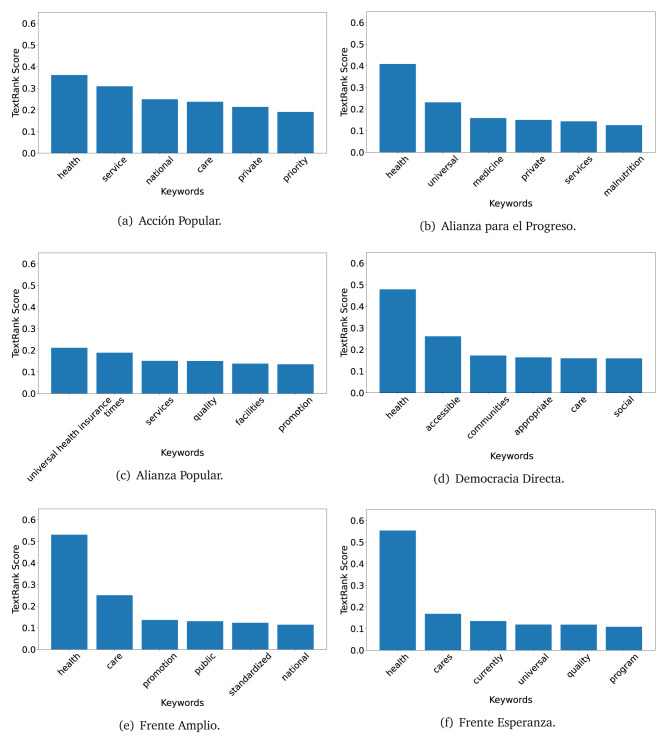
TextRank results, keywords and scores, for government plans in 2016 (political parties one through six).

**Figure 5.**
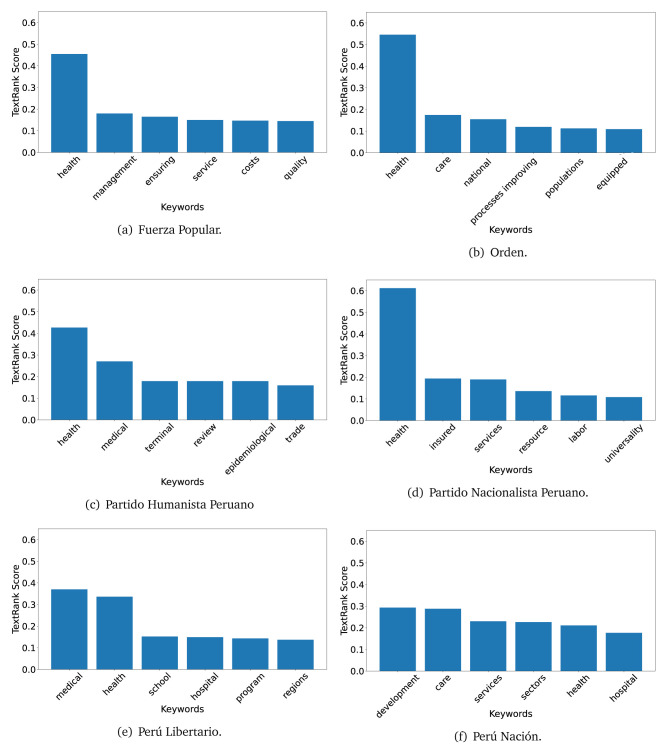
TextRank results, keywords and scores, for government plans in 2016 (political parties seven through twelve).

**Figure 6.**
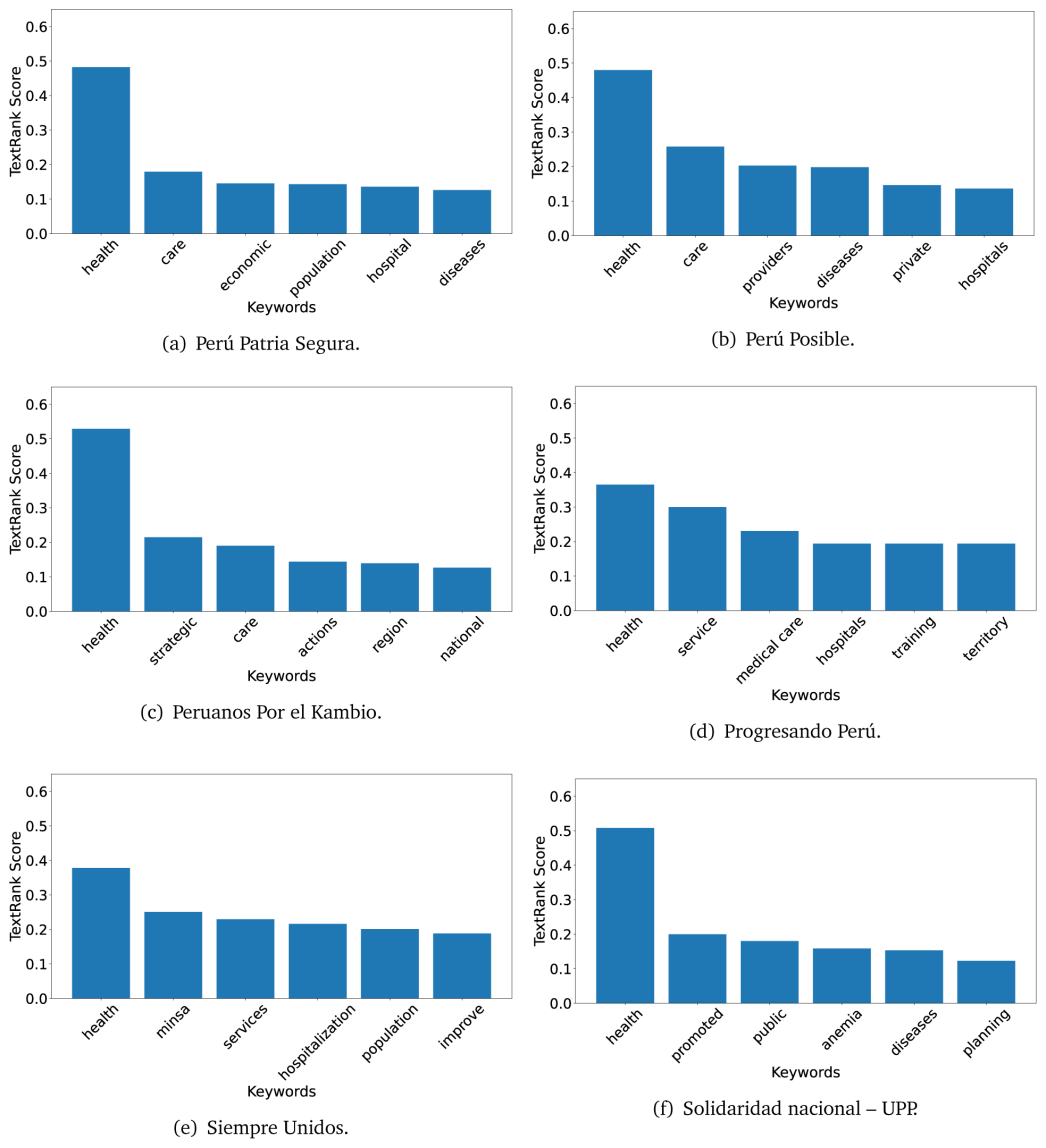
TextRank results, keywords and scores, for government plans in 2016 (political parties thirteen through eighteen).

**Figure 7.**
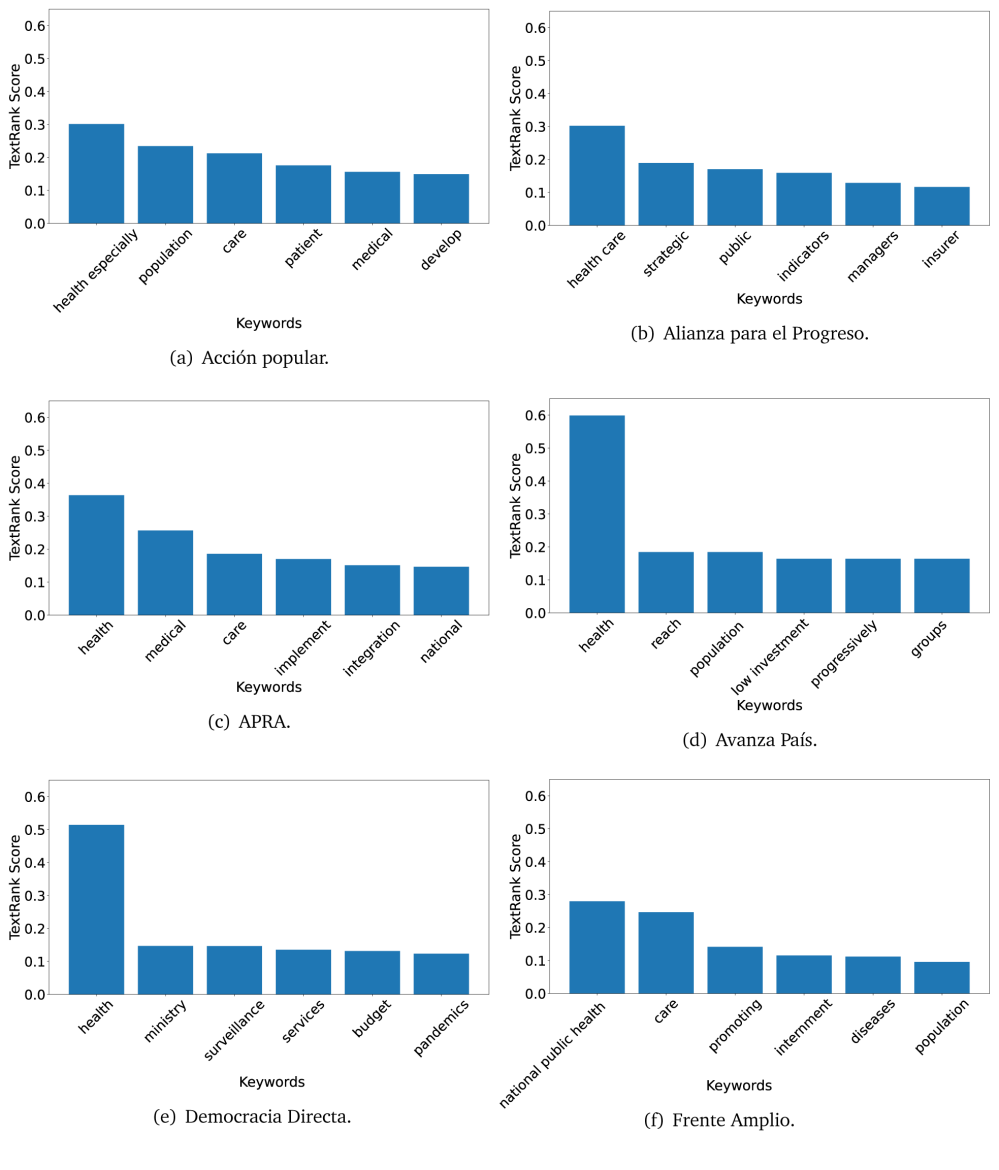
TextRank results, keywords and scores, for government plans in 2021 (political parties one through six).

**Figure 8.**
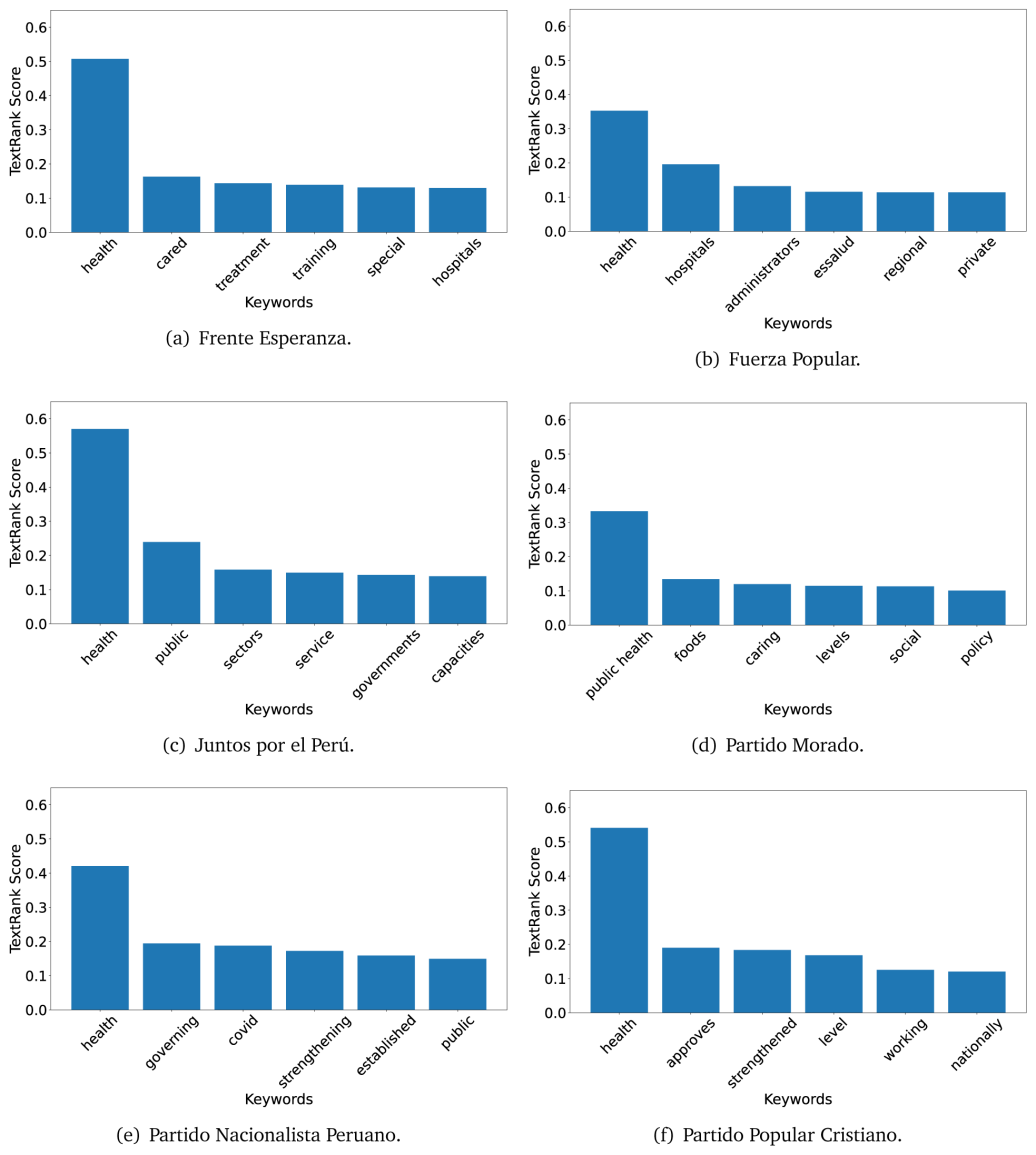
TextRank results, keywords and scores, for government plans in 2021 (political parties seven through twelve).

**Figure 9.**
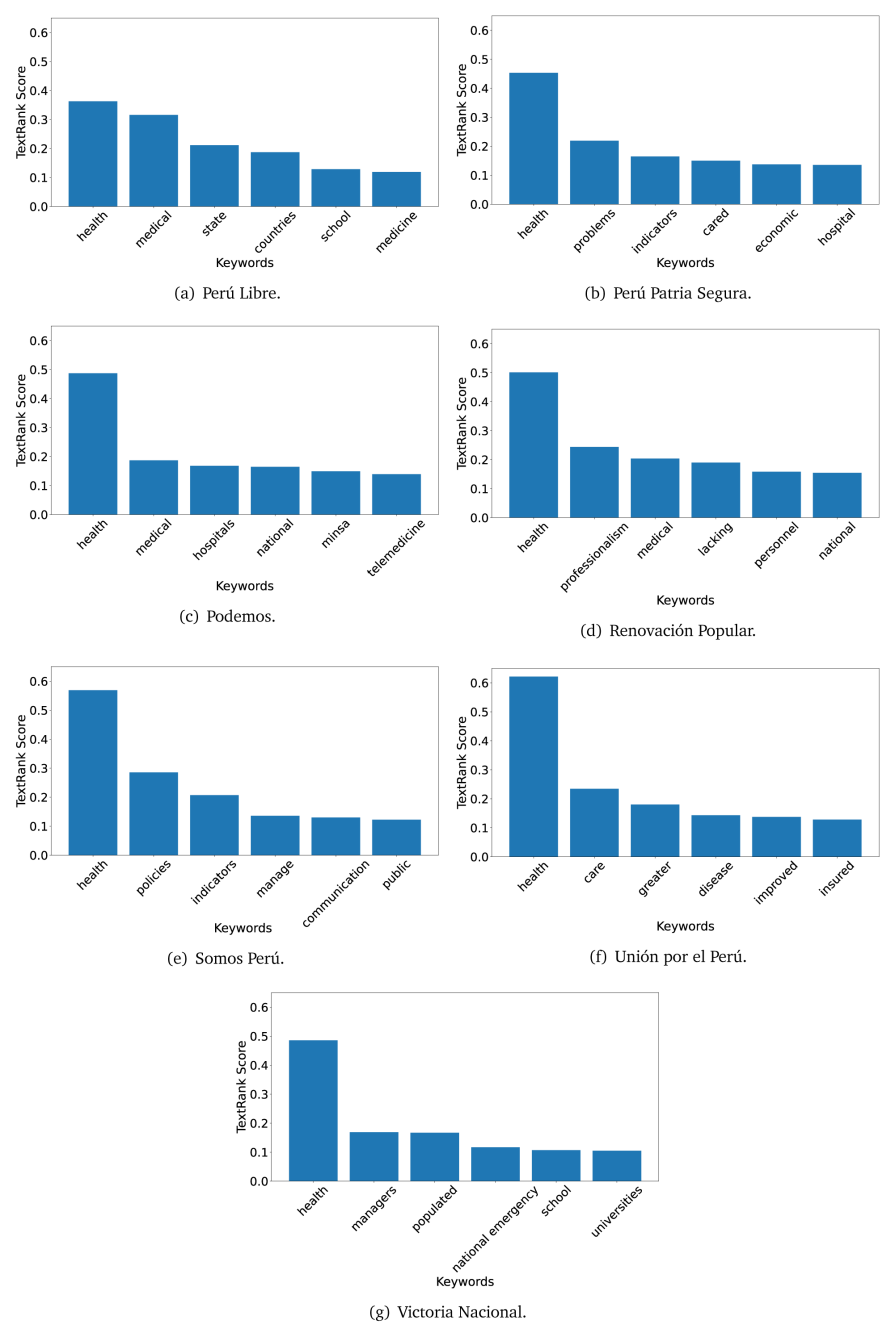
TextRank results, keywords and scores, for government plans in 2021 (political parties thirteen through nineteen).

In both 2016 (see Figure 4, Figure 5 and Figure 6) and 2021 (see Figure 7, Figure 8 and Figure 9), the term ’health’ was the most frequent keyword. In 2016, terms regarding ’universal health coverage’ were also frequent; in 2021 however, terms about ’universal health coverage’ were not present. In 2016, the terms were more general, and appeared to focus on improving the health system with words like ’hospital’, ’population’, ’public’ or ’region’.

In 2021, we found words relevant in the context of the COVID-19 pandemic, like ’national emergency’. We also found words that reflected the characteristics of the health system at the beginning of the COVID-19 pandemic, for example ’low investment’ and ’lacking’.

Bear in mind that although TextRank provides the most frequent keywords, these words alone do not provide insights about the context of the text. Therefore, it becomes relevant to study the context of the text, so we also used the KeyBERT (group of terms) and Rake (to study phrases) algorithms.

### KeyBERT

**Figure 10.**
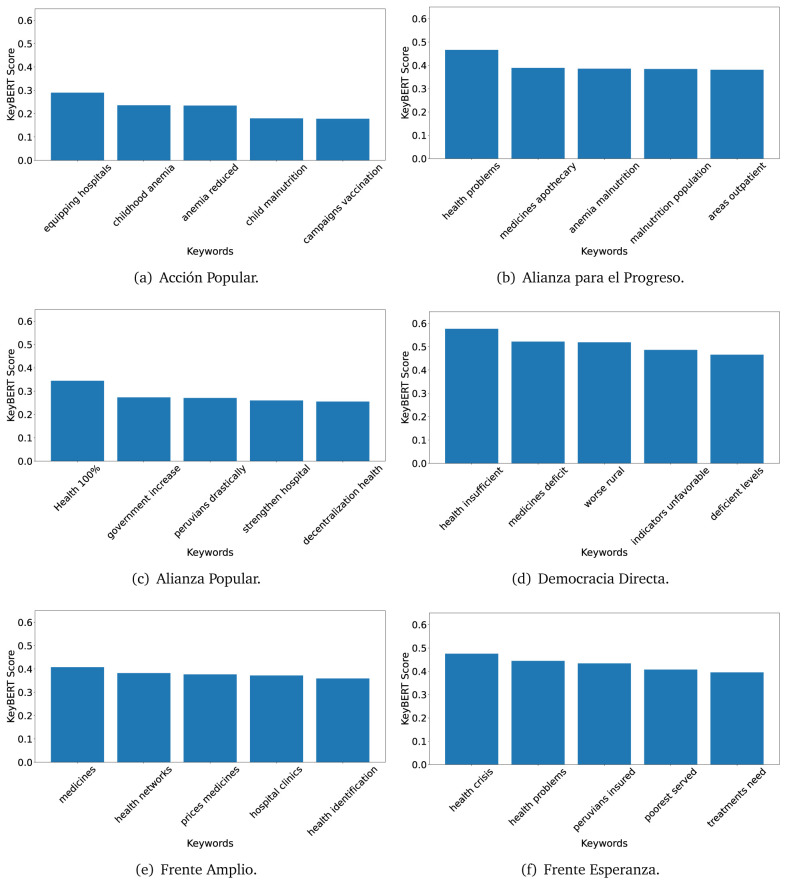
KeyBERT results, keywords, and scores, for government plans in 2016 (political parties one through six).

**Figure 11.**
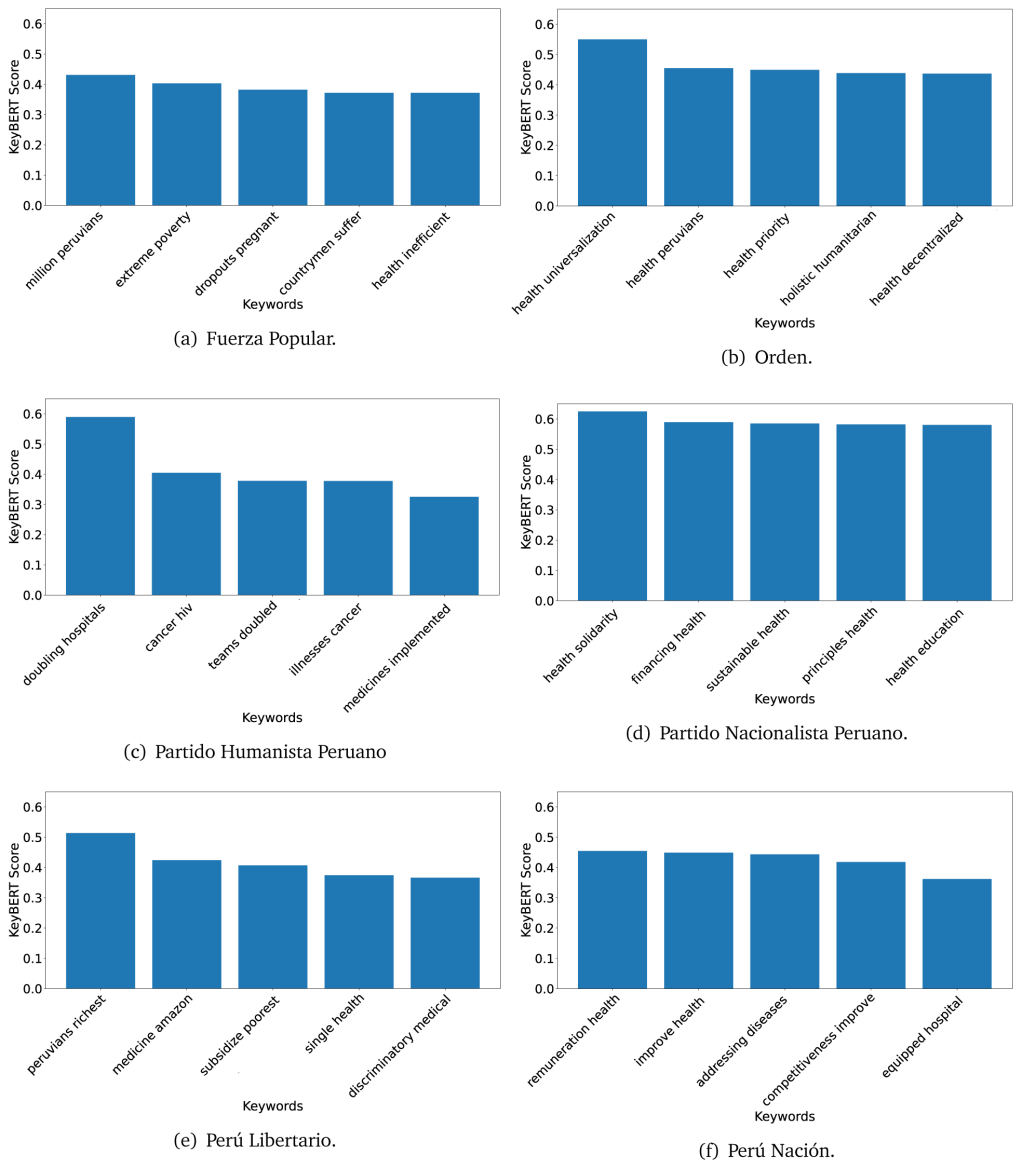
KeyBERT results, keywords, and scores, for government plans in 2016 (political parties seven through twelve).

**Figure 12.**
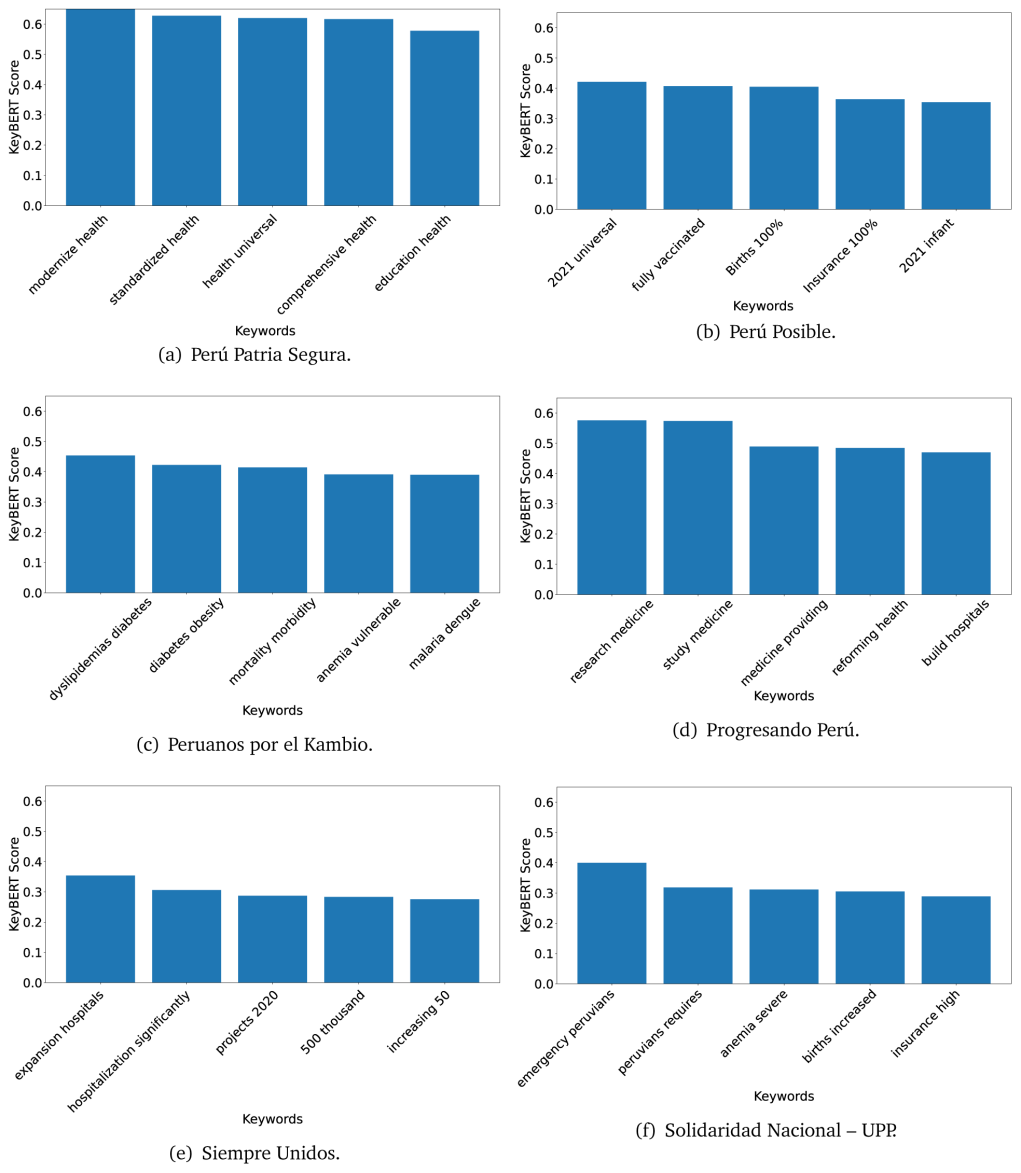
KeyBERT results, keywords, and scores, for government plans in 2016 (political parties thirteen through eighteen).

**Figure 13.**
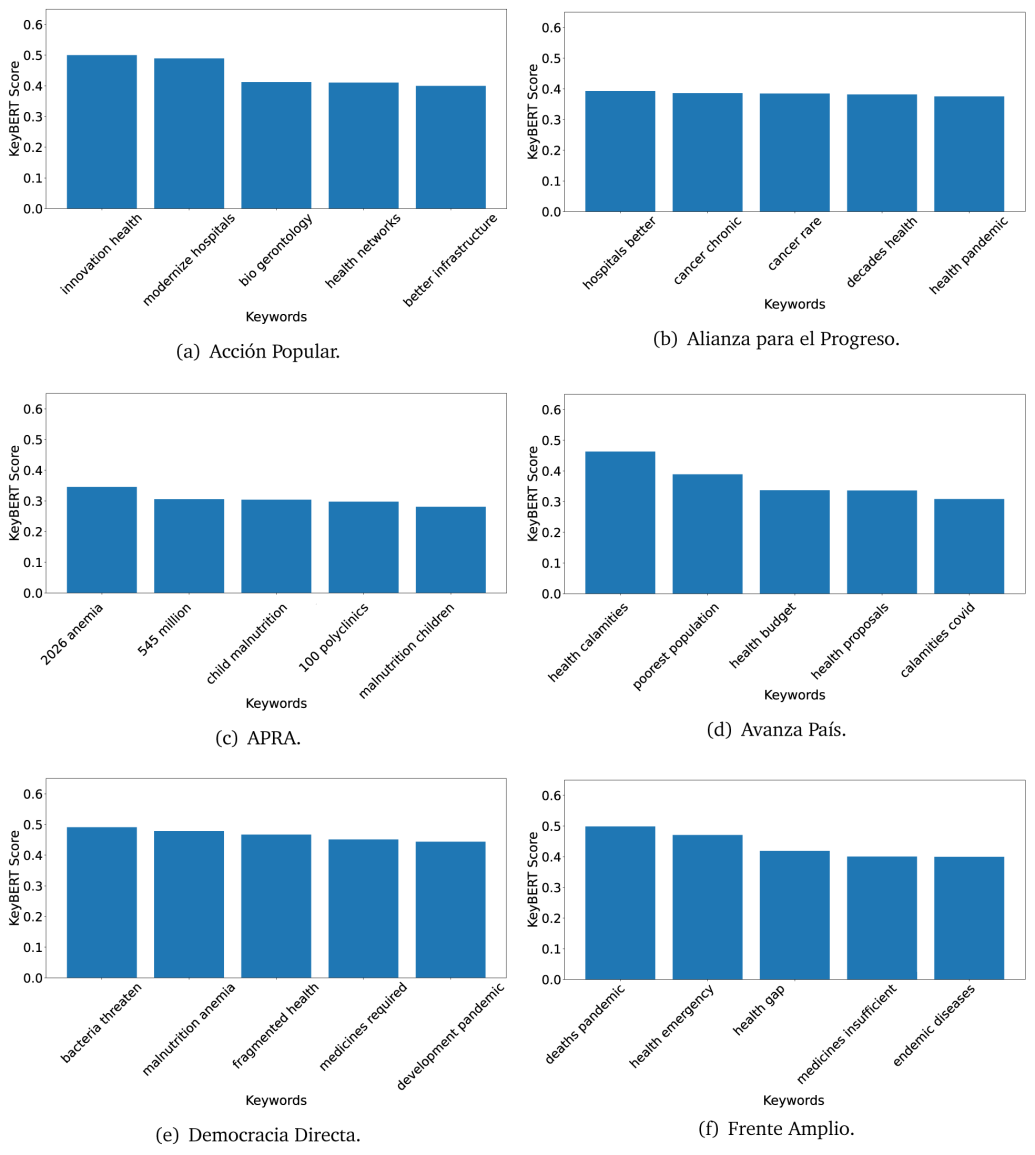
KeyBERT results, keywords, and scores, for government plans in 2021 (political parties one through six).

**Figure 14.**
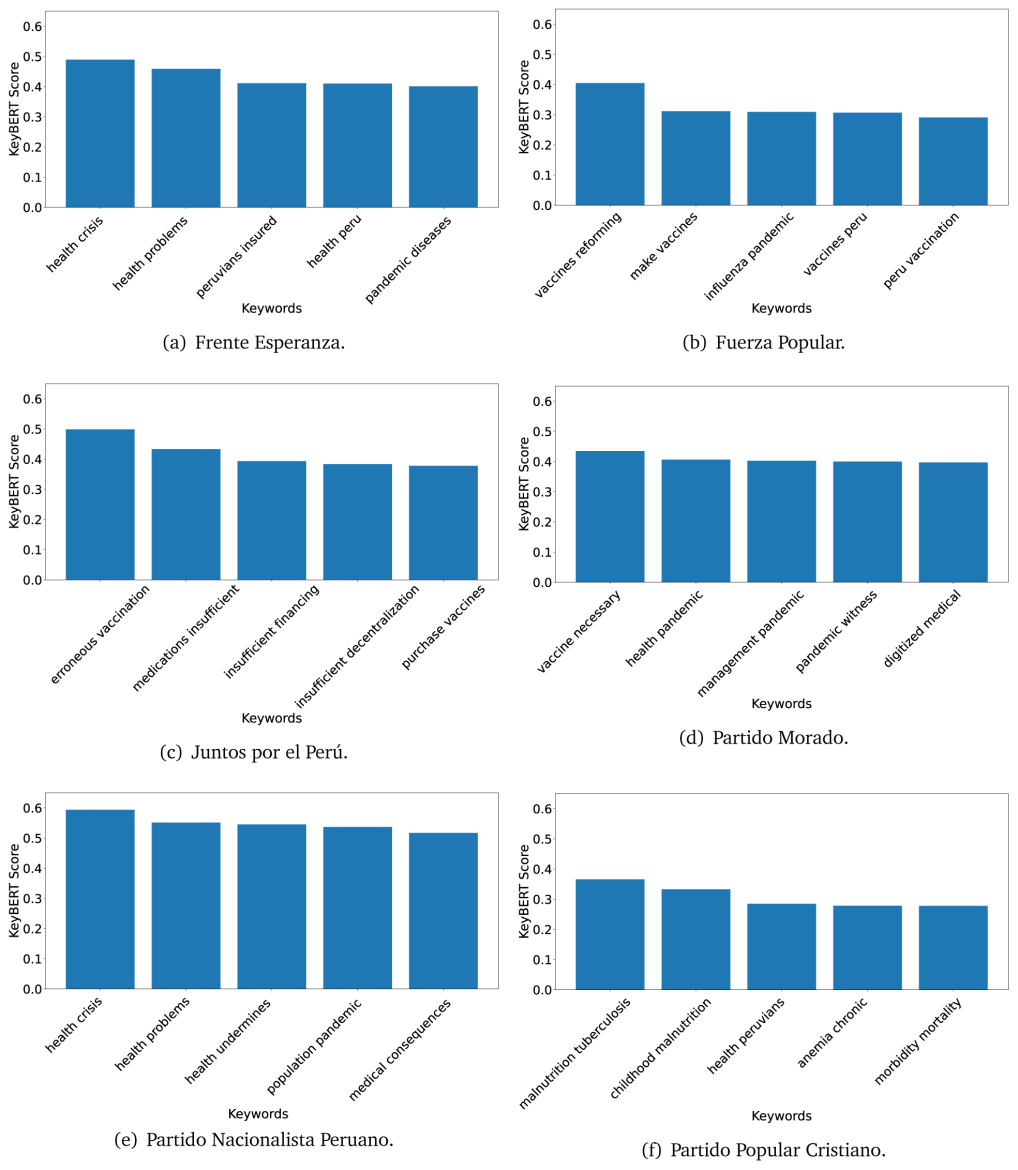
KeyBERT results, keywords, and scores, for government plans in 2021 (political parties seven through twelve).

**Figure 15.**
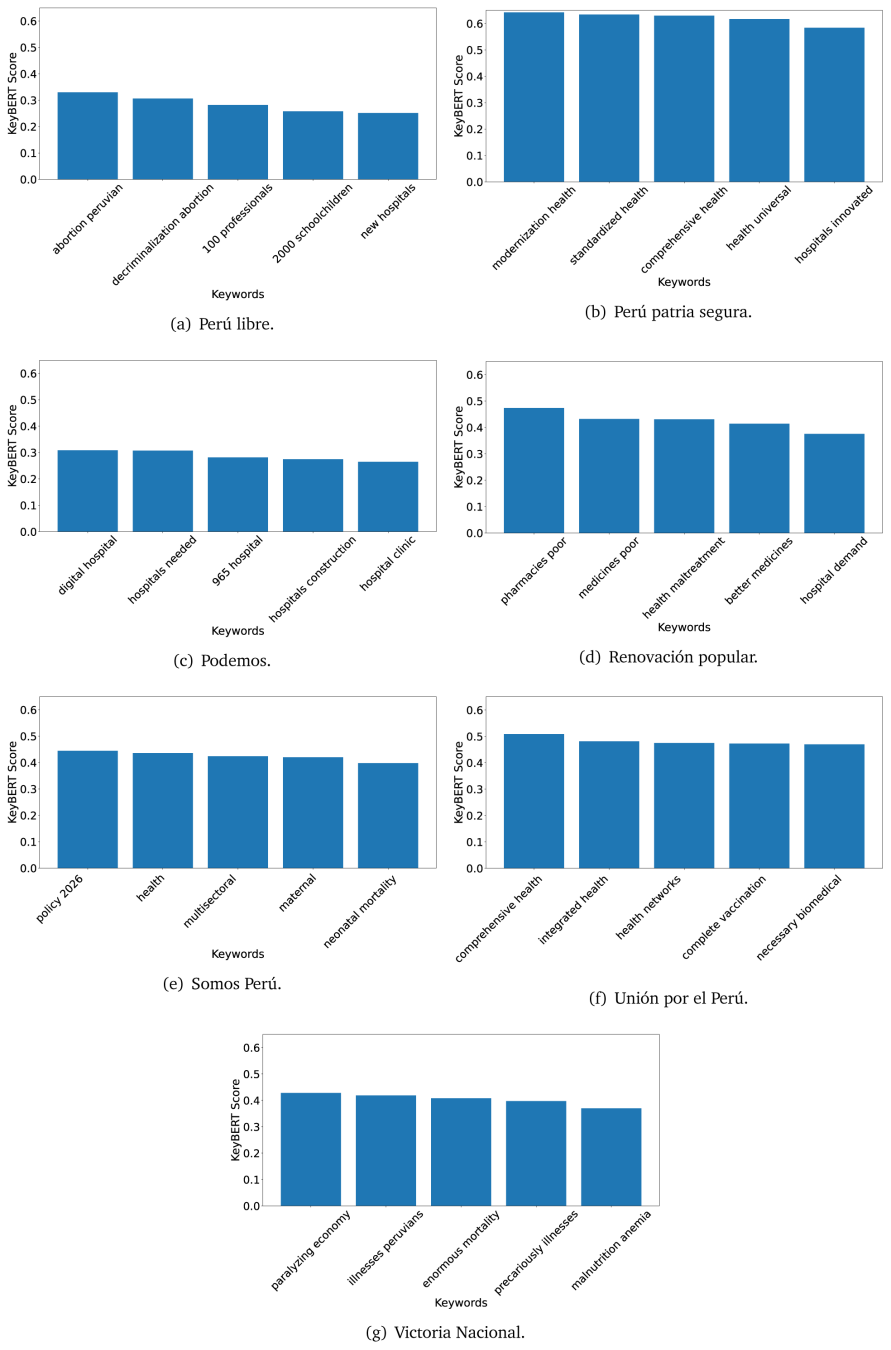
KeyBERT results, keywords, and scores, for government plans in 2021 (political parties thirteen through nineteen).

TextRank ^17^ and KeyBERT ^30^ provides keywords (between one and three words), but the latter uses a variation of the BERT algorithm to chose keywords based on the context of the text. Moreover, KeyBERT shows the main keywords with their score. For this algorithm, the five main keywords have been extracted using the same government plans, i.e., 2016 (see Figure 10, Figure 11 and Figure 12) and 2021 (see Figure 13, Figure 14 and Figure 15).

Consistent with what we observed using the TextRank algorithm, the 2016 (see Figure 10, Figure 11 and Figure 12) keywords appeared to be more general. The terms talked about, for example, ’health solidarity’, ’health crisis’, and ’health inefficient’. However, the KeyBERT algorithm did find other keywords which revealed more concise concepts: ’campaign vaccination’, ’equipped hospital’ and ’health education’. For one government plan (see Figure 12(c)), all keywords were about diseases: ’dyslipidaemias diabetes’, ’diabetes obesity’, ’mortality morbidity’, ’anemia vulnerable’, and ’malaria dengue’. Furthermore, the keywords retrieved with the KeyBERT algorithm also revealed underlying characteristics of the political party. For example (see Figure, 11(e)), the keywords of a left-wing political party were ’Peruvian richest’, ’subsidise poorest’ and ’discriminatory medical’.

As also pointed out with the TextRank algorithm, in 2021 (see Figure 13, Figure 14 and Figure 15) the keywords appeared to be more concrete (e.g., ’health networks’, ’Peruvians insured’ and ’hospitals needed’) and also covered topics related with the COVID-19 pandemic (e.g., ’health pandemic’, ’management pandemic’ and ’eaths pandemic’). In one government plan (see Figure 14(b)), four out of five keywords were about vaccination: ’vaccines reforming’, ’make vaccines’, ’vaccines Peru’, and ’Peru vaccination’. Similarly, other government plan had four keywords about diseases: ’malnutrition tuberculosis’, ’childhood malnutrition’, ’anemia chronic’ and ’morbidity mortality’.

### Rake

For visualisation purposes, we chose the top five key phrases per government plan in 2016 (see Table 3) and 2021 (see Table 4).

**Table 3.** List of top five Rake key phrases for government plans in 2016.

Political Party	Keywords Phrases
Acción Popular	’national agreement child malnutrition’, ’health plan agreed upon’, ’24 hours per day’, ’drinking water service coverage’, ’universal service coverage’
Alianza para el progreso	’apothecary modules installed inside warehouses’, ’multiple minsa health service providers’, ’health service providers’, ’quality generic medicines’, ’promoting universal insurance’
Alianza popular	’drastically reduce chronic child malnutrition’, ’remuneration reform’, generating mechanisms’, ’single health information management system’, ’expand universal health insurance throughout’
Democracia directa	’culturally appropriate health service delivery system’, ’integrated health care based’, ’health care model centered’, ’public health system’, ’traditionally excluded groups’
Frente amplio	’private health system meets required standards regulated’, ’mental health centers creation’, ’health system deficient mental health system’, ’health needs territorialize health care’, ’national health information system modernize’
Frente esperanza	’must reach 2 beds per thousand inhabitants’, ’1000 USD per year per inhabitant’, ’subsidising brand name drugs’, ’also faces serious problems’
Fuerza popular	’respecting compulsory licensing rules promote access’, ’accompanying multidisciplinary community health teams’, ’seguro integral de salud’ (national health system), ’ensuring better working conditions’, ’sustainable development goal 2’
Orden	’mobile health units’, ’health must assume full responsibility’, ’establishing free mobile health units’, ’50 million soles’, ’rational comprehensive national health plan’
Partido humanista peruano	’preventive health services full coverage’, ’healthy person without access’, ’free trade agreement signed’, ’single patient without care’, ’life total health plan’
Partido nacionalista peruano	’one thousand seven hundred million soles’, ’deadlines progressive decentralization sustainability surveillance’, ’approximately four hundred million soles’, ’universal insurance revolve around coverage’, ’total peruvian population target 100’
Perú libertario	’achieving total free public health’, ’17 billion dollars annually’, ’abolish discriminatory medical care’, ’entire educational revolution must’, ’state must set fees’
Perú nación	’developing properly equipped hospital centers’, ’develop public policies aimed’, ’health services currently provided’, ’health care bonds’, ’health care personnel’
Perú patria segura	’health programs communicable disease programs fully strengthened’, ’economic status marginal populations virtually unattended’, ’high quality management system inefficient remuneration’, ’centers managing excellent health services number’, ’std prevention programs well strengthened’
Perú posible	’intangible health solidarity fund’, ’elderly extend mental health care’, ’mass communication strategies throughout’, ’more health plan peru’, ’provide free primary care’
Peruanos por el kambio	’generating three intervention programs per year’, ’human resources directorate, regional governments’, ’moving towards universal health coverage’, ’least 4 annual programs focused’, ’regional governments strategic guideline
Progresando Perú	’contemporary scientific medical knowledge’, ’reforming health care services’, ’localised health care’, ’intercultural health program’, ’high mortality rate’
Siempre unidos	’achieving one hospital per 500 thousand inhabitants’, ’hospitalisation significantly improve emergency services encourage’, ’state must provide budgetary support’, ’reduce patient waiting time’, ’current 3rd level hospitals’
Solidaridad nacional -UPP	’25 per 1000 live births’, ’online id card key’, ’offer family planning services’, ’promote healthy living habits’, ’higher infant mortality rates’

**Table 4.** List of top five Rake key phrases for government plans in 2021.

Political Party	Keywords Phrases
Acción Popular	’equipped primary care medical posts’, ’technologically innovate office services’, ’basic health plan appropriate’, ’allow solving health problems’, ’reduce health care gaps’
Alianza para el progreso	’tier health insurance fund administrative institution’, ’several parallel systems creates many problems’, ’high cost fund strategic’, ’universal insurance system strategic’, ’health care centers
APRA	’health professionals per inhabitant according’, ’integrated medical information management system’, ’articulated territorial assistance network’, ’universal digital medical record system’, ’articulated territorial assistance networks’
Avanza país	’latin american average levels’, ’health services continues’, ’efficient health strategies’, ’public health services’, ’promote citizen participation’
Democracia directa	’properly equipped isolation areas within’, ’budget program “0131 control” ’, ’promotes healthy lifestyle habits’, ’health sector receives approximately’, ’mental health problems attended’
Frente amplio	’private health system meets required standards’, ’comprehensive care obligations using political’, ’implementing integrated health networks open’, ’national health care plan approved’, ’comprehensive health career policy throughout’
Frente esperanza	’prepare adequately trained health professionals’, ’also faces serious problems’, ’clearly identify pandemic diseases’, ’must reach 2 beds’, ’health personnel widely trained’
Fuerza popular	’subsidized insurance regime must guarantee equal benefits’, ’longer build temporary medical care centers’, ’3 thousand new detected per day’, ’approximately one million rapid serological tests’, ’advance towards universal social security’
Juntos por el Perú	’facilitates severe administrative sanctions regulatory framework’, ’public system implemented covid sequelae treatment program’, ’single public health system implemented national central’, ’intersectoral action regional governments assume responsibility’, ’operation public pharmacy chains operating’,
Partido morado	’reinforce said “integration”’, rapid test occurs around 20 days’, ’integrality: public networks must talk’, ’mainly use rapid tests’, ’regional health directorates must give way’
Partido nacionalista peruano	’200 properly equipped provincial epidemiology offices’, ’32 certified biosafety level iii laboratories’, ’people receiving mental health services establish’, ’private sector must work together’, ’high complexity national service works’,
Partido popular cristiano	’gender approach national teaching articulation system service created resolution act’, ’competency strengthening programs implemented people development plans approved multidisciplinary health teams’, ’cost disease fund high cost disease fund created nationally approved’, ’proven health spending efficiency monitoring system resolution act definition’, ’adequate working conditions approved health career document resolutive act’
Perú libre	’would surely end discriminatory medical care’, ’expanding universal public health coverage’, ’163 million usd per year’, ’peruvian legislation currently contemplates’, ’regional medical resident program’
Perú patria segura	’std prevention programs well strengthened reducing cases’, ’goal communicable disease programs fully strengthened’, ’economic condition marginalised populations practically lacking’, ’achieve strengthen communicable disease programs’, ’marginalised populations practically lacking’
Podemos	’four unrelated subsystems coexist — confusingly —’, ’health science popular soup kitchens repowering’, ’minsa promote comprehensive health insurance transfer’, ’precarious since 379 health establishments’, ’1000 health establishments nationwide creation’
Renovación Popular	’national continuous professional qualification module’, ’national health promotion plan’, ’health sector create centers’, ’health sector centers’, ’inadequate public management’
Somos Perú	’standardised electronic medical record’, ’healthy country whose paradigms’, ’community mental health centers throughout’, ’health financing must include health promotion’, ’implement community mental health centers’
Unión por el Perú	’acute respiratory infections ari would’, ’200 million new soles’, ’eliminate chronic child malnutrition’, ’2020 covid 19 pandemic’, ’single integrated health system’
Victoria nacional	’electronic medical records implemented 100%’, ’yet achieved universal health insurance’, ’electronic medical records’, ’effective universal health insurance system’, ’130 deaths per million’

Therefore, in 2016, a topic that was recurrent in four government plans was ’universal health coverage’. When the phrases addressed specific diseases/conditions, these were malnutrition, mental health, communicable diseases, and sexually transmitted diseases (see Table 3).

On the other hand, in 2021, ’universal health coverage’ was also a frequent topic found in phrases of four government plans. Mental health also appeared often (in three government plans). There were also phrases related to COVID-19, and the fact that the health system in Peru is fragmented (see Table 4).

## Discussion

### Main findings

We used novel techniques (NLP) to study the health chapters of the government plans of the political parties participating in the 2016 (18 plans) and 2021 (19 plans) general elections in Peru. 

The TF-IDF algorithm revealed that the 2021 government plans repeated more terms; in 2021 there were 43 terms with a frequency at or above 0.10, whereas this threshold was met by nine terms in the 2016 government plans. This could suggest that in 2021 these documents elaborated more on some topics using the same vocabulary.

The LDA analysis defined two groups: one gathering words signalling things the population would receive (e.g., ’insurance’), and the other with terms about the health system (e.g., ’capacity’). The LDA analysis also assigned government plans to either group. This suggested whether a government plan would focus on delivering goods or services to the population, whereas others would focus on improving the health system.

The TextRank revealed some key words. Interestingly, the keyword phrase ’universal health coverage’ were frequent in 2016, but this did not appear in 2021, when keywords about the COVID-19 pandemic (e.g., ’national emergency’) and the limited capacities of the health system (e.g., ’low investment’) were frequent. This provides preliminary evidence on how the main focus of the government plans shifted between 2016 and 2021.

The KeyBERT analysis revealed keywords, but these were learnt based on the context of the text. Interestingly, these keywords provided more insight about the underlying profile of the political party. This suggests that it would be possible to identify characteristics of the writer (in this case of the political party) based on the text and its content.

The Rake analysis provided key phrases, instead of keywords, and the results provided more insight about the documents. For example, this algorithm revealed that phrases ’universal health coverage’ appeared both in 2016 and 2021. Similarly, phrases with “mental health” were found in both years. Phrases about COVID-19 were only found in 2021.

### Interpretation and potential explanations

The first finding was that the dataset with the 2021 government plans was larger than the 2016 dataset. Similarly, the TF-IDF analysis also showed that the number of terms with a frequency of ≥0.10 was 4.8-fold higher in 2021 than in 2016. This could imply that health has gained more relevance over the last five years, particularly since 2020 when the COVID-19 pandemic revealed the limitations and deficiencies of the health systems in Peru.

The TF-IDF analysis suggested different underlying approaches between the 2016 and 2021 government plans. Our hypothesis is that the 2016 plans were more general, because frequent terms were ’agreed’, ’agreement’, ’political’ and ’plan’; overall, these terms would suggest a non-specific approach. Conversely, in the 2021 government plans, words like ’bonus’, ’gerontology’, and ’migration’, were often found and would suggest specific proposals or subjects. ’Bonus’ are very related with the ongoing epidemiological scenario, in which the government has given economic bonuses to the population. ’Gerontology’ could be a response to the ageing population, and the health problems that come along. Finally, ’migration’ could be a response to the overwhelming migration phenomenon in Peru and South America, where countries are receiving people from Venezuela. Our hypothesis is that the frequent terms in 2021 suggested more specific proposals than those in 2016.

The LDA analysis provided two groups, and also showed to which group each government plan is most similar. Our hypothesis is that Group 0 included terms or things the population would receive or directly interact with, and Group 1 included terms or things that the healthcare provider would deliver. In 2016, the LDA analysis suggested that most of the government plans would belong to Group 1; this could imply that the 2016 government plans focused on how to improve the services they provide or deliver. In 2021, there were even more government plans highly likely to belong to Group 1, probably because the COVID-19 pandemic made them realise the urgent need for structural changes to improve the health system.

As pointed out before with the LDA algorithm, the TextRank analysis also showed that keywords in 2016 were more general than in 2021, when we also found keywords addressing the COVID-19 pandemic. Interestingly, in 2021 the TextRank analysis also revealed a critique view of the health system at the beginning of the COVID-19 pandemic. This suggests that some government plans were not only about proposing or offering new programs or interventions, but they also evaluated the health system beforehand, ideally to inform their proposals, making these as specific as possible to solve the main problems or limitations of the health system.

Even though TextRank and KeyBERT would deliver keywords, the keywords obtained with KeyBERT gave more context about the government plan or the political party. Government plans in which most of the keywords obtained with KeyBERT were about diseases, could suggest that they will focused on these illness, their risk factors and consequences; in other words, the proposals could be disease-specific. Similarly, government plans in which many of the keywords were about vaccination -in 2021 presumably regarding COVID-19-, could suggest that their priority would be to get vaccines, and deliver these to the best of their ability; it could also suggest that they will focus –mostly– on the pandemic, while other problems would be addressed in parallel or with less priority. Finally, in accordance with the fact that the KeyBERT algorithm finds keywords based on the context of the text, this algorithm gave insights about the underlying characteristics of the political party. This algorithm could be used to dissect the profile of the political party.

The Rake analysis provided key phrases that the unsupervised model could compose from the original texts. Both in 2016 and 2021, a recurrent topic was universal health coverage, signalling that this was a hot topic which, apparently, has not been solved since 2016. In this line, the 2021 phrases addressed the fragmentation of the healthcare system in Peru, where several systems coexist (e.g., private, public, social security, and military forces) ^31^. This fragmentation has challenged, and sometimes limited, the response to the COVID-19 pandemic. Finally, mental health was addressed both in 2016 and 2021. This implies that the concern about the mental health of the population has risen since 2016. Also, this suggests that the improvement achieved so far still needs further work to secure optimal mental health for the population. Communicable diseases were also mentioned in both 2016 and 2021; however, non-communicable diseases were absent from these phrases, despite the large burden they impose on the population and health systems ^32^.

### Strengths and limitations

We used novel techniques to study the health chapters of the government plans of the 2016 and 2021 Peruvian presidential elections. In so doing, we provided novel insights about these documents to better inform practitioners, researchers, public health experts, and the general population in Peru. This exercise can be replicated in other countries hosting elections this year or soon. We also hope to have sparked interest in NLP techniques to strengthen public health and health policy research, which have lots of data sources which can benefit from NLP analysis.

There are, however, limitations we must acknowledge. First, we focused on the health chapter/section of each government plan which surely contained much of the information for the health sector. Nevertheless, we cannot completely rule out that other chapters could have included additional information about their health-related plans; for example, the chapter/section about economics could have addressed the budget for the health sector or plans of investments. We argued that the most relevant health information must have been included in the health chapters/sections herein analysed, so that arguments outside these chapters would not substantially change our findings or conclusions. Second, we used the government plans as they were, and translated them into English. To the best of our knowledge, the most used NLP algorithms are only available for texts in English. We acknowledge that some words/terms may not have had the ideal translation from Spanish to English through Google Translator; however, this potential limitation affecting single words may have not biased the overall findings and conclusions. We hope that this work sparks interest in the Spanish-speaking scientific community to use NLP into clinical medicine and public health research, while also developing NLP algorithms in Spanish. Third, the analyses and interpretations were mostly data-driven, and should be interpreted in that context. This work was not designed to be a comprehensive political, anthropological, or linguistic scrutiny of the 2016 and 2021 government plans in Peru, rather the application of novel artificial intelligence techniques to further expand the understanding of the health chapters in these government plans. Fourth, we did not compare the government plan of the same political party in 2016 and 2021. A prospective easement of the political parties was beyond the scope of this work, and such exercise would not be possible for all parties.

## Conclusion

This NLP analysis of the health chapters of government plans for the general elections in Peru in 2016 and 2021, showed that NLP are a useful tool to dissect these documents in terms of keywords and phrases based on frequency and context. The NLP analysis could inform about the underlying priorities or main subjects addressed by each government plan, while also revealing the profile of the political parties. NLP analysis could also be included in the research of health policies and politics during general elections, and could provide informative summaries for the general population.

## Data availability

### Underlying data

Figshare: Data - Government plans in the 2016 and 2021 Peruvian presidential elections: A natural language processing analysis of the health chapters. https://doi.org/10.6084/m9.figshare.14466699.v1 ^33^.

The project contains the following underlying data:

- planesSalud 2016.csv (dataset where each row is a sentence of the health chapter in each government plan. For example, if the government plan ‘ABC’ has a health chapter with 15 sentences, there will be 15 rows (each containing a sentence))
- planesSalud 2021.csv (dataset where each row is a sentence of the health chapter in each government plan. For example, if the government plan ‘ABC’ has a health chapter with 15 sentences, there will be 15 rows (each containing a sentence))
- planesSalud Comp 2016.csv (dataset where each row is a health chapter in each government plan. There will be as many rows as government plans, each row containing the health chapter in full)
- planesSalud Comp 2021.csv (dataset where each row is a health chapter in each government plan. There will be as many rows as government plans, each row containing the health chapter in full)

Data are available under the terms of the Creative Commons Attribution 4.0 International license (CC-BY 4.0). 
