# Supplementary material for: Government plans in the 2016 and 2021 Peruvian presidential elections: A natural language processing analysis of the health chapters
Source: Wellcome Open Res. 2022 Oct 25;6:177. Originally published 2021 Jul 8. [Version 5] doi: 10.12688/wellcomeopenres.16867.5 (PMC11809155; doi:10.12688/wellcomeopenres.16867.5)
Supplement: Peer review history

# Reviewer report: Reviewer response for version 5

**DOI:** 10.21956/wellcomeopenres.20483.r115207

**Published:** 2025-01-28

**Authors:** Kiran Khatter

**Affiliations:** 1 BML Munjal University, Gurgaon, India

This paper presents a study on the application of Natural Language Processing (NLP) algorithms to analyze the health chapters of government plans from the 2016 and 2021 Peruvian presidential elections. The study addresses a gap in the use of NLP for health policy analysis, moving beyond traditional applications like electronic health records or social media analysis. This expands the scope of NLP applications in public health. The study compares different NLP algorithms to extract and analyze keywords and phrases, aiming to highlight underlying themes and priorities in health policy during these election cycles.

- The discussion on the practical applications of the findings is brief. More concrete examples of how the insights could inform health policy, public health strategies, or electoral decision-making would strengthen the paper's impact.
- The use of Google Translate for data translation could introduce biases. This limitation should be addressed with more detail in the paper, including potential effects on findings and how these were mitigated.
- The paper lacks sufficient detail on the preprocessing steps, feature extraction, and validation for the datasets used. Clear descriptions of these processes would enhance reproducibility.
- The rationale for selecting the specific NLP algorithms is underexplored. Including a justification for their suitability over alternative approaches and a discussion of their comparative effectiveness would add depth.
- The paper acknowledges limitations but could provide a more detailed exploration, including the impact of dataset size variability, algorithmic constraints (e.g., LDA’s fixed groupings), and generalizability to other countries or contexts.
- The qualitative implications of findings, such as insights into political party priorities or public health challenges, could be expanded. Incorporating more context-based analysis would make the results more impactful.
- The writing could be improved for clarity and conciseness. Some sections are overly technical and may not be easily accessible to non-specialist readers. Defining key terms and providing examples would improve comprehension.

Is the work clearly and accurately presented and does it cite the current literature?

Partly

If applicable, is the statistical analysis and its interpretation appropriate?

Partly

Are all the source data underlying the results available to ensure full reproducibility?

Yes

Is the study design appropriate and is the work technically sound?

Partly

Are the conclusions drawn adequately supported by the results?

Yes

Are sufficient details of methods and analysis provided to allow replication by others?

Yes

Reviewer Expertise:

Image Processing, NLP, Blockchain, Fuzzy/Neutrosophic sets

I confirm that I have read this submission and believe that I have an appropriate level of expertise to confirm that it is of an acceptable scientific standard, however I have significant reservations, as outlined above.

# Reviewer report: Reviewer response for version 5

**DOI:** 10.21956/wellcomeopenres.20483.r116415

**Published:** 2025-01-10

**Authors:** Andria Arisal

**Affiliations:** 1 National Research and Innovation Agency, Jakarta, Indonesia

This manuscript effectively demonstrates how NLP techniques can be applied to extract important healthcare-related topics in political documents, aiding the public in comparing priority topics across political parties. The analysis is comprehensive and well-executed.

 However, the reviewer suggests the following improvements to enhance the clarity, methodological rigor, and impact of the manuscript:

 1. Move the discussion of the manuscript's contribution from the Discussion section to the Introduction to better frame the study's significance upfront.

 2. Clearly explain the differences between TextRank and KeyBERT in the methods section and provide an argument for using one over the other. Or, if the authors want to use both, include a head-to-head comparison (e.g., in a single side-by-side figure) to better contextualize their results. Avoid spreading their results across separate sets of figures (e.g., Figures 4-9 for TextRank and Figures 10-15 for KeyBERT).

 3. Remove “health*” as a keyword from TextRank and KeyBERT analyses, as it is a ubiquitous term in the dataset and does not provide additional insight into distinguishing topics.

 4. The current number of bar charts is overwhelming and diverts the reader’s attention from the core discussion. Consider more integrative and comparative visualization techniques, such as:

    - Word clustering with intersections among political parties to highlight shared priorities.

    - LDAvis for visualizing group classifications in LDA, offering a more intuitive understanding of topic distributions.

    - Combine Figure 1 into a grouped bar chart to display the 2016 and 2021 data series together.

    - Present a grouped bar chart showing terms with the highest aggregate TextRank or KeyBERT scores across political parties to emphasize shared interests.

 6. The authors chose two clusters for the LDA analysis but did not justify this decision. Including coherence scores or perplexity metrics to determine the optimal number of clusters would improve methodological rigor.

 If possible the authors could also:

 1. Add statistical tests to validate observed trends and differences between 2016 and 2021, such as tests for significant differences in keyword frequencies or topic distributions across years.

 2. The imbalance in dataset size between 2016 and 2021 likely influences results. Normalize keyword frequencies and clustering probabilities to account for differences in the volume of data.

 3. The discussion could benefit from more actionable insights. For example, explain how these findings could influence public health policy or decision-making in Peru and other similar contexts.

 4. Address ethical considerations around using government plans for NLP analysis, such as potential implications of misinterpretation or misrepresentation of political priorities.

Is the work clearly and accurately presented and does it cite the current literature?

Yes

If applicable, is the statistical analysis and its interpretation appropriate?

Yes

Are all the source data underlying the results available to ensure full reproducibility?

Partly

Is the study design appropriate and is the work technically sound?

Partly

Are the conclusions drawn adequately supported by the results?

Yes

Are sufficient details of methods and analysis provided to allow replication by others?

Partly

Reviewer Expertise:

Data Analytics, Natural Language Processing, High Performance Computing

I confirm that I have read this submission and believe that I have an appropriate level of expertise to confirm that it is of an acceptable scientific standard, however I have significant reservations, as outlined above.

# Reviewer report: Reviewer response for version 5

**DOI:** 10.21956/wellcomeopenres.20483.r113436

**Published:** 2025-01-07

**Authors:** Shaina Raza

**Affiliations:** 1 Vector Institute, Toronto, Ontario, Canada

The paper presents an important problem, however, I would like authors to mention where is there contributions, is it methods or problem they solved?

 I see methods are quite simplistic? is it planned? Can they include some analysis through named entity recognition as in recent clinal NLP and NER methods, or they can use some LLMs (free ones) to get some insights to make it better.

 It depends what authors are trying to convey, their computational method or solving health NLP.

Is the work clearly and accurately presented and does it cite the current literature?

Partly

If applicable, is the statistical analysis and its interpretation appropriate?

Yes

Are all the source data underlying the results available to ensure full reproducibility?

Partly

Is the study design appropriate and is the work technically sound?

Yes

Are the conclusions drawn adequately supported by the results?

Partly

Are sufficient details of methods and analysis provided to allow replication by others?

Partly

Reviewer Expertise:

NLP, LLMs

I confirm that I have read this submission and believe that I have an appropriate level of expertise to confirm that it is of an acceptable scientific standard, however I have significant reservations, as outlined above.

# Reviewer report: Reviewer response for version 4

**DOI:** 10.21956/wellcomeopenres.19477.r48856

**Published:** 2022-10-14

**Authors:** Ayoub Bagheri

**Affiliations:** 1 Utrecht University, Utrecht, The Netherlands

Hereby I am submitting my reviews on the paper entitled “Government plans in the 2016 and 2021 Peruvian presidential elections: A natural language processing analysis of the health chapters.” The paper is well written and while the topic is of much interest, there are some serious concerns regarding the methods, analysis, and experimental design of the paper. Below are comments in detail:

- Please provide a link to your toolkit as it is mentioned that it is available to use for other researchers.
- Could you elaborate on the contributions of this work?
- I would like to know why the authors choose these methods and how do they compare them with each other.
- Is it an improvement if you are using a lemmatisation method in TF-IDF and LDA?
- What is the reasoning when you cluster data into only two groups in LDA? It would be nice if you look at 5, 10, 20, and 50 as well.
- Could you explain in the paper what is the input to the LDA model?
- What are the limitations of using KeyBERT? Do you have to use fixed vectors?
- Could you provide the equation for the rake method and describe what was the library you used for this?
- If you look at the frequencies, words similar to 'health' should appear in most of the documents, therefore with TF-IDF they should gain a very low weight. How can you respond to this argument? Isn't 'health' a stopword in this analysis?
- In Table 2, would you provide the probabilities for each term in groups as well?
- How do you compare Fig. 2b and Fig. 3b?
- The keywords for Fig. 4c are not clear.
- From page 8 until 26 you just see figures and tables with no text explaining them. 
- The first point in the discussion is about applying novel techniques in this study. I must say that I do not agree with the authors as I do not see any novel method in the paper. The paper does not propose any novelty (that, of course, is not the focus of the study and the journal), while except KeyBERT (2020), the other methods are quite used in many studies: TF-IDF (1972), LDA (2003), Rake (2010), textrank (2004).
- Simply because you are looking at the health chapters, TF-IDF should remove the term 'health' from the analysis, unless you say that many of the documents do not contain the word health.

Is the work clearly and accurately presented and does it cite the current literature?

Yes

If applicable, is the statistical analysis and its interpretation appropriate?

Partly

Are all the source data underlying the results available to ensure full reproducibility?

Partly

Is the study design appropriate and is the work technically sound?

Partly

Are the conclusions drawn adequately supported by the results?

Yes

Are sufficient details of methods and analysis provided to allow replication by others?

No

Reviewer Expertise:

Natural Language Processing, Data Science

I confirm that I have read this submission and believe that I have an appropriate level of expertise to confirm that it is of an acceptable scientific standard, however I have significant reservations, as outlined above.

# Response

**Published:** 2022-10-16

**Authors:** Rodrigo M Carrillo-Larco

**Affiliations:** Imperial College London, UK

We are thankful for the insightful comments by the reviewer. We appreciate the time he invested in reading our work. Please see our point-by-point answers below. We hope these satisfy the reviewer’s expectations.

**C1. Please provide a link to your toolkit as it is mentioned that it is available to use for other researchers.**

 R1. We have uploaded the Jupyter Notebooks for these analyses to the Figshare repository reported in the Data Availability statement, which was updated accordingly. The sentence in the introduction about the ‘toolkit’ was removed.

**C2. Could you elaborate on the contributions of this work?**

 R2. Yes, the contribution of this work has been further elaborated in the Introduction and Discussion sections. The new lines read:

 [introduction] *Our work contributes to the NLP health literature by leveraging on standard and well-known algorithms to analyse health policy/politics documents. Because most previous NLP health research focused on electronic health records (i.e., clinical oriented) or social media (i.e., population health oriented), our work expanded the use of NLP in health research to new documents (policy/politics documents) with potential public health implications. Furthermore, another contribution of our work also lies in the fact that we used a sort of text documents that, arguably, may be available to a much broader audience. Even though electronic health records and social media are valuable sources of information for NLP analyses, electronic health records may be seldom available in low- and middle-income countries where people could also engage less in social medial. Nevertheless, in most countries during election period there may be some sort of documents summarizing the proposals by the candidates who may share these as much as possible for electors to familiarize themselves with the candidates.*

 [discussion] *Finally, the contribution of this work lies in the fact that we expanded the data sources frequently used in NLP health research, which has mostly leveraged on texts from electronic health records and social media. Unfortunately, electronic health records may not be available in many low- and middle-income countries, where people may not engage as much in some social media outlets as their peers in high-income countries. Thus, our work may democratize the use of NLP in health research by leveraging on a new data source (e.g., materials distributed during national elections) that, arguably, is accessible to a much broader population. Future work, moving from electronic health records and social media, could focus on texts from newspapers and open-access documents published by authorities such as reports from government officers, summaries from government meetings, and summaries of debates at the congress; again, all these sources may be available to a large population above and beyond electronic health records and social media.*

**C3. I would like to know why the authors choose these methods and how do they compare them with each other.**

 R3. We have provided this information in the Methods section, right before the description of the algorithms. The new lines read:

*We used five NLP algorithms: TF-IDF, LDA, TextRank, KeyBERT, and Rake. We chose these algorithms because they are frequently used in the NLP literature, they are available through most libraries in Python and R facilitating the replication and outreach of our work, and they satisfied our goals (i.e., to describe the health chapters in government plans *by summarising words and terms *).*

*TF-IDF and TextRank reveals keywords; TF-IDF does so based on the relative frequency of each term whereas TextRank selects keywords based on a trained model. Compared to TF-IDF and TextRank, KeyBERT as well as Rake reveal keywords or short phrases (e.g., 3-5 words) based on trained models. LDA, on the other hand, groups texts into unknown clusters and the user labels such clusters (i.e., as in unsupervised machine learning with K-means). Altogether, these algorithms provide a framework to describe the health chapters in government plans going from unique terms (TF-IDF and TextRank), to short phrases (KeyBERT and Rake), and grouping into unknown clusters (LDA).*

**C4. Is it an improvement if you are using a lemmatisation method in TF-IDF and LDA?**

 R4. We followed the standard recommendations for TF-IDF and LDA as shown in references 25, 26, and 27 in the manuscript. For both algorithms, lemmatisation was suggested before implementing them.

**C5. What is the reasoning when you cluster data into only two groups in LDA? It would be nice if you look at 5, 10, 20, and 50 as well.**

 R5. We have justified our choice of two (02) groups in LDA. The new lines read (new text underlined):

*For this work, we defined two groups (i.e., we specified that the LDA algorithm should create two groups following an unsupervised approach from the data). Two is the absolute minimum number of groups and we chose such option this being an exploratory work compounded with other NLP algorithms, and we did not focus solely on the LDA results. For a discussion on this limitation, please refer to the Strengths and Limitations section.*

 We also discussed this decision, specifically in Strengths and Limitations section:

*Sixth, the LDA algorithm was applied with the absolute minimum number of groups (i.e., to identify two clusters). We could have tried to find 5, 10, 20 or more groups; however, dividing the texts in many thin groups would have not been ideal. First, we only worked with the health chapters which were, on average, one or two pages long; in other words, these chapters did not have much content to be dissected into many clusters. Had we analysed the full plans (not only the health chapters), finding more groups would have helped to separate all sections. Second, the government plans were concise straight-to-the-point documents (sometime bullet points); that is, they did not cover several topics nor in detail. This made it difficult to dissect the texts into many thin groups.*

**C6. Could you explain in the paper what is the input to the LDA model?**

 R6. We added these lines:

*The input for training the LDA algorithm was all the government plans (separately for each year), having removed the stop words and lemmatized the terms. Then, with the cleaned (lemmatized and without stop words) text for each political party, the model assigns each party to a group defined during the LDA training.*

**C7. What are the limitations of using KeyBERT? Do you have to use fixed vectors?**

 R7. The question was not very clear to us. We apologise unreservedly and hope this answer addresses the issue. We added these lines in the Methods section where we described the KeybERT analysis:

*A limitation of this algorithm is that it retrieves a maximum of six keywords; though this did now affect our work because we sought five keywords as it was the case for other algorithms.*

**C8. Could you provide the equation for the rake method and describe what was the library you used for this?**

 R8. We added these lines:

*We used the class Rake from the rake_nltk library; for further details including the equation for the Rake method please refer to reference 15.*

**C9. If you look at the frequencies, words similar to 'health' should appear in most of the documents, therefore with TF-IDF they should gain a very low weight. How can you respond to this argument? Isn't 'health' a stopword in this analysis?**

 R9. We have included the term ‘health’ as a stop word (i.e., excluded the term ‘health’ from the TF-IDF analysis). The TF-IDF figures and results have been updated accordingly. Notably, the main results did not change.

 Following this recommendation, and because TF-IDF has the same pre-processing as the LDA algorithm, the term ‘health’ was also excluded from the LDA analysis. We updated the results and table accordingly.

 The term ‘health’ was not removed from the other analyses because they use the semantic context of the text and removing a word would go against this recommendation. Moreover, removing the word ‘health’ could change some relevant key words or short phrases such as ‘public health’, ‘universal health’, or ‘health insurance’.

**C10. In Table 2, would you provide the probabilities for each term in groups as well?**

 R10. Table 2 now includes the probabilities for each of the ten terms. Please, keep in mind that the terms have changed as well (not the same as in the original submission), because the LDA analysis was re-ran having removed the word ‘health’ (i.e., ‘health’ is now a stop word).

**C11. How do you compare Fig. 2b and Fig. 3b?**

 R11. We have included these lines in the Results section, at the end of the LDA sub-heading.

*Figure 2 shows the LDA results for the government plans in 2016 and Figure 3 for the plans in 2021. While these figures show that political parties are more inclined to either Group 0 or Group 1, a direct comparison between 2016 and 2021 is troublesome because: i) the same exact political parties did not participate in the two elections; ii) the composition of the political parties in terms of roles, interests and priorities may have changed between 2016 and 2021; and iii) amidst the COVID-19 pandemic, the focus of the health-related government plans would be different between 2016 and 2021. Perhaps in response to the COVID-19 pandemic, the difference observed between Figure 2B and Figure 3B could signal that the 2021 plans focused more on terms related to the structure of the health system (i.e., Group 1 appeared to cluster terms that are related with the structure of the health system).*

**C12. The keywords for Fig. 4c are not clear.**

 R12. Unfortunately, we could not customize the very first label for Figure 4C. The very first label was a three-word term which font/size we could not customize without compromising the other labels. Even though we tried different methods to change that label only, we could not find an optimal solution. We apologise for the inconvenience. Happily, the message and interpretation of Figure 4C was not altered.

**C13. From page 8 until 26 you just see figures and tables with no text explaining them.**

 R13. The text explaining or describing the figures and tables are just before the pages containing only figures and table. This was the layout decided by the journal’s typesetters for the PDF file. However, the HTML version of the manuscript has the figures and tables embedded in the text. We apologise for the inconvenience and we will work with the editors to improve the layout of the PDF version.

**C14. The first point in the discussion is about applying novel techniques in this study. I must say that I do not agree with the authors as I do not see any novel method in the paper. The paper does not propose any novelty (that, of course, is not the focus of the study and the journal), while except KeyBERT (2020), the other methods are quite used in many studies: TF-IDF (1972), LDA (2003), Rake (2010), textrank (2004).**

 R14. Indeed. We appreciate the discrepancy and the opportunity to clarify. We modified the very first sentence in the Discussion (new text underlined):

*We used standard and well-known NLP algorithms* *to study health chapters of the government plans of the political parties in the 2016 (18 plans) and 2021 (19 plans) general elections in Peru. We did not aim to develop new NLP algorithms nor to modify the available algorithms, rather, we aimed to apply available NLP algorithms in a novel topic: health policy/politics.*

**C15. Simply because you are looking at the health chapters, TF-IDF should remove the term 'health' from the analysis, unless you say that many of the documents do not contain the word health.**

 R15. The word ‘health’ was removed from the TF-IDF and LDA analyses (i.e., it was set as a stop word). The results, figures, and tables for the TF-IDF and LDA analyses were updated accordingly.

# Reviewer report: Reviewer response for version 4

**DOI:** 10.21956/wellcomeopenres.19477.r48096

**Published:** 2022-01-21

**Authors:** Glen Dario Rodriguez Rafael

**Affiliations:** 1 Unidad de Postgrado de la Facultad de Ingenieria de Informàtica y Sistemas, Universidad Nacional Mayor de San Marcos, Lima, Peru

Thank you for the improvements. The article now is fit to be indexed.

Is the work clearly and accurately presented and does it cite the current literature?

Yes

If applicable, is the statistical analysis and its interpretation appropriate?

Partly

Are all the source data underlying the results available to ensure full reproducibility?

Yes

Is the study design appropriate and is the work technically sound?

Yes

Are the conclusions drawn adequately supported by the results?

Partly

Are sufficient details of methods and analysis provided to allow replication by others?

Yes

Reviewer Expertise:

AI, machine learning, data mining, text mining

I confirm that I have read this submission and believe that I have an appropriate level of expertise to confirm that it is of an acceptable scientific standard.

# Reviewer report: Reviewer response for version 3

**DOI:** 10.21956/wellcomeopenres.19368.r47709

**Published:** 2022-01-11

**Authors:** Glen Dario Rodriguez Rafael

**Affiliations:** 1 Unidad de Postgrado de la Facultad de Ingenieria de Informàtica y Sistemas, Universidad Nacional Mayor de San Marcos, Lima, Peru

In the last review, I asked the authors to exclude some words from the TF-IDF analysis, and update figure 1. The authors did it.

 But the authors are using the previous analysis (analysis in the previous version without words excluded) in the parragraph starting with "The TF-IDF analysis suggested different underlying approaches between the 2016 and 2021 government plans. Our hypothesis is that the 2016 plans were more general, because frequent terms were ’agreed’, ’agreement’, ’political’ and ’plan’; overall, these terms would suggest a non-specific approach. "...

 Please review all the text (not only that paragraph) in order to assure that you are not presenting the results of the old analysis.

Is the work clearly and accurately presented and does it cite the current literature?

Yes

If applicable, is the statistical analysis and its interpretation appropriate?

Partly

Are all the source data underlying the results available to ensure full reproducibility?

Yes

Is the study design appropriate and is the work technically sound?

Yes

Are the conclusions drawn adequately supported by the results?

Partly

Are sufficient details of methods and analysis provided to allow replication by others?

Yes

Reviewer Expertise:

AI, machine learning, data mining, text mining

I confirm that I have read this submission and believe that I have an appropriate level of expertise to confirm that it is of an acceptable scientific standard, however I have significant reservations, as outlined above.

# Response

**Published:** 2022-01-13

**Authors:** Rodrigo M Carrillo-Larco

**Affiliations:** Imperial College London, UK

Thank you very much for pointing this out. Two paragraphs in the discussion have been modified accordingly.

# Reviewer report: Reviewer response for version 2

**DOI:** 10.21956/wellcomeopenres.19245.r47104

**Published:** 2021-12-07

**Authors:** Glen Dario Rodriguez Rafael

**Affiliations:** 1 Unidad de Postgrado de la Facultad de Ingenieria de Informàtica y Sistemas, Universidad Nacional Mayor de San Marcos, Lima, Peru

I thanks the author for their efforts in improving this paper. I have only one observation standing:

 Regarding Q2 of first review, "change"; and "execute" and "force" have not been excluded in Figures 1(b) and 1(a), so I am not sure you excluded them for both years. I mentioned 3 words as examples of filler wordls, but there are more words without intrinsic "policy" meaning: based, force, agreed, execute, addressed, exempting, promote.

 A filler world would be any common adjective or verb (based, agreed, addressed are adjective, execute, promote, exempt-ing are verbs) that can be used for many purposes; and some nouns (force).

Is the work clearly and accurately presented and does it cite the current literature?

Yes

If applicable, is the statistical analysis and its interpretation appropriate?

Partly

Are all the source data underlying the results available to ensure full reproducibility?

Yes

Is the study design appropriate and is the work technically sound?

Yes

Are the conclusions drawn adequately supported by the results?

Partly

Are sufficient details of methods and analysis provided to allow replication by others?

Yes

Reviewer Expertise:

AI, machine learning, data mining, text mining

I confirm that I have read this submission and believe that I have an appropriate level of expertise to confirm that it is of an acceptable scientific standard, however I have significant reservations, as outlined above.

# Response

**Published:** 2021-12-09

**Authors:** Rodrigo M Carrillo-Larco

**Affiliations:** Imperial College London, UK

We appreciate the positive feedback by the reviewer. We apologise for the confusion when uploading and publishing the updated figures. We have excluded the following words: based, force, agreed, execute, addressed, exempting, promote lower, bio, isc, expected and trend. The text and figures have been updated accordingly.

# Reviewer report: Reviewer response for version 1

**DOI:** 10.21956/wellcomeopenres.18605.r46096

**Published:** 2021-11-11

**Authors:** Glen Dario Rodriguez Rafael

**Affiliations:** 1 Unidad de Postgrado de la Facultad de Ingenieria de Informàtica y Sistemas, Universidad Nacional Mayor de San Marcos, Lima, Peru

I think this article is an interesting exploration on the topic of policy analysis using NLP. However, there are some issues that need to be addressed:

- Regarding the statistical analysis, the text of the health chapters has a large variance in length and, for many political parties, is quite small. There is no discussion of how this fact could affect the conclusions drawn using NLP.
- TF-IDF as used here is of scarce value, because "filler words"were not excluded (example: execute, force, change). These are no traditional filler words, but in the context of political discourse, they are filler words.
- LDA analysis is interesting and worthy.
- Discussion of KeyBERT analysis is too brief. Please discuss further.
- The problem with NLP for Spanish text is not the lack of algorithms, it is the lack of datasets (text corpus, both POS tagged and untagged, annotated, etc.)
- A couple of references that would be useful to add:
  Leeson, W., Resnick, A., Alexander, D., & Rovers, J. (2019). Natural Language Processing (NLP) in qualitative public health research: a proof of concept study. International Journal of Qualitative Methods, 18. ^1^
  Katre, P. D. NLP Based Text Analytics and Visualization of Political Speeches. International Journal of Recent Technology and Engineering 8(3):8574-8579 ^2^

Is the work clearly and accurately presented and does it cite the current literature?

Yes

If applicable, is the statistical analysis and its interpretation appropriate?

Partly

Are all the source data underlying the results available to ensure full reproducibility?

Yes

Is the study design appropriate and is the work technically sound?

Yes

Are the conclusions drawn adequately supported by the results?

Partly

Are sufficient details of methods and analysis provided to allow replication by others?

Yes

Reviewer Expertise:

AI, machine learning, data mining, text mining

I confirm that I have read this submission and believe that I have an appropriate level of expertise to confirm that it is of an acceptable scientific standard, however I have significant reservations, as outlined above.

## References

[1] : Natural Language Processing (NLP) in Qualitative Public Health Research: A Proof of Concept Study. *International Journal of Qualitative Methods* .2019;18: 10.1177/1609406919887021 10.1177/1609406919887021

[2] : NLP Based Text Analytics and Visualization ofPolitical Speeches. *International Journal of Recent Technology and Engineering* .2019;8(3) : 10.35940/ijrte.C6503.098319 10.35940/ijrte.C6503.098319

# Response

**Published:** 2021-11-13

**Authors:** Rodrigo M Carrillo-Larco

**Affiliations:** Imperial College London, UK

**Q1. Regarding the statistical analysis, the text of the health chapters has a large variance in length and, for many political parties, is quite small. There is no discussion of how this fact could affect the conclusions drawn using NLP.**

 R1. This has been discussed in the limitations section: “ *Fifth, the text of the health chapters varied in length. The LDA analysis was trained in a dataset with all the government plans together. Government plans with longer text, hence with more words and information, could have driven the algorithm results; conversely, short government plans could have provided less information to the LDA algorithm. For transparency, we reported the length of each government plan and the results should be interpreted considering this potential limitation.”*

**Q2. TF-IDF as used here is of scarce value, because "filler words"were not excluded (example: execute, force, change). These are no traditional filler words, but in the context of political discourse, they are filler words.**

 R2. These “filler words” (execute, force and change) were excluded from the analysis. The methods section has been updated accordingly: “ *The following filler words were excluded: execute, force and change; although these are not traditional filler words, in the political context they are filler words.*” Also, the TF-IDF results were updated and so were the figures (figure 1).

**Q3. LDA analysis is interesting and worthy.**

 R3. We appreciate the positive feedback.

**Q4. Discussion of KeyBERT analysis is too brief. Please discuss further.**

 R4. In the discussion, in the sub-section ‘ *Interpretation and potential explanations*’, the second-to-last paragraph has been modified to discuss further.

**Q5. The problem with NLP for Spanish text is not the lack of algorithms, it is the lack of datasets (text corpus, both POS tagged and untagged, annotated, etc.)**

 R5.  This has been included in the limitations section: “ *In addition to more algorithms in Spanish, the scientific community should also secure datasets in Spanish which currently lack to train available and new NLP models. Where possible, these datasets of text in Spanish (and other languages) should be tagged and untagged, as well as annotated or annotated*.”

**Q6. A couple of references that would be useful to add:**

**Leeson, W., Resnick, A., Alexander, D., & Rovers, J. (2019). Natural Language Processing (NLP) in qualitative public health research: a proof of concept study. International Journal of Qualitative Methods, 18.1**

**Katre, P. D. NLP Based Text Analytics and Visualization of Political Speeches. International Journal of Recent Technology and Engineering 8(3):8574-85792**

 R6. These two references were included. The first reference was included at the end of this sentence, next to a reference we already had: “ *On the other hand, public health research appears not to have benefited from NLP algorithms, despite the fact that this research field also has large data sources of text, such as reports and policy briefs.*” The second reference was included at the end of this new sentence in the limitations section: “ *This work was not designed to be a comprehensive political (though NLP is also useful for studying political speech) …*”
